# Next-generation biodiesel production: catalytic innovations and scalable microalgal biorefinery systems

**DOI:** 10.1039/d6ra02860h

**Published:** 2026-07-03

**Authors:** Mohamed Hemdan, Yasser Elbahloul, Peter F. Farag, Wael S. El-Sayed

**Affiliations:** a School of Biotechnology, Badr University in Cairo (BUC) Badr City Cairo 11829 Egypt Mohamed.Hemdan@buc.edu.eg; b Department of Biotechnology, Faculty of Biotechnology, Sinai University-Kantara Branch Ismailia 41636 Egypt; c Department of Botany and Microbiology, Faculty of Science, Alexandria University Alexandria Egypt; d Department of Microbiology, Faculty of Science, Ain Shams University Cairo 11566 Egypt

## Abstract

The transition toward low-carbon liquid fuels is increasingly urgent due to fossil fuel depletion, carbon budget overshoot, and the escalating environmental and public health impacts associated with combustion-based energy systems. Biodiesel has emerged as a strategically important renewable fuel owing to its compatibility with existing diesel infrastructure and its potential to deliver meaningful life-cycle greenhouse gas reductions. This review provides a comprehensive and critical assessment of next-generation biodiesel production, integrating catalytic science, feedstock development, process engineering, sustainability metrics, and industrial implementation perspectives within a unified framework. Fundamental aspects of biodiesel chemistry, fuel properties, and quality standards are first outlined to establish a rigorous basis for subsequent analysis. The evolution of biodiesel feedstocks from first-generation edible oils to second-generation waste-derived lipids and third-generation microalgae is critically examined, highlighting sustainability trade-offs, resource constraints, and scalability considerations associated with each feedstock class. Particular attention is devoted to microalgal biorefinery systems, which offer exceptional lipid productivity, non-arable land utilization, carbon dioxide capture potential, and opportunities for resource recovery, while simultaneously facing significant cultivation, harvesting, and downstream processing challenges. The review further provides a mechanistic and comparative evaluation of homogeneous, heterogeneous, bifunctional, and enzymatic catalytic systems, together with quantitative assessment of catalytic performance, feedstock tolerance, operational stability, and industrial applicability. Recent advances in process-intensification technologies, including supercritical processing, continuous-flow reactors, reactive distillation, and non-thermal enhancement techniques, are critically evaluated in the context of biodiesel commercialization. Furthermore, techno-economic analysis (TEA), life-cycle assessment (LCA), technology-readiness considerations, and industrial scalability challenges are integrated to identify key bottlenecks and future development priorities. By integrating catalytic innovation, feedstock evolution, process-intensification technologies, sustainability assessment, and commercialization perspectives, this review provides a distinctive roadmap for translating laboratory-scale advances into commercially competitive, low-carbon biodiesel technologies capable of supporting future energy-transition objectives.

## Abstract 2

From feedstock evolution to industrial deployment, next-generation biodiesel technologies combine advanced catalysis, intensified processing, and sustainable biorefinery systems.

## Graphical abstract

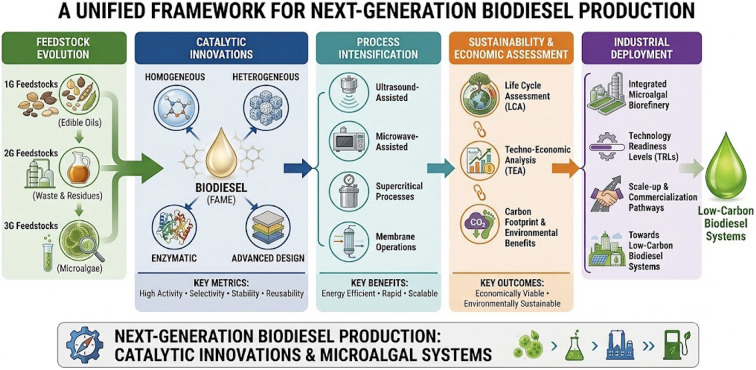

## 1. Introduction

The global energy system is undergoing a critical transition driven by steadily increasing energy demand, the finite nature of fossil fuel resources, and escalating environmental and societal pressures. Although renewable electricity generation has expanded significantly, liquid fuels remain indispensable for energy-intensive sectors such as heavy-duty transportation, aviation, maritime shipping, and remote power generation.^1,2^ These sectors continue to depend primarily on fossil fuels due to their high energy density, reliability, and compatibility with existing infrastructure. However, the long-term sustainability of this dependence is increasingly uncertain as conventional reserves decline, extraction shifts toward more complex and costly resources, and energy markets remain exposed to geopolitical instability.^3,4^ Collectively, these factors highlight the urgent need for alternative liquid fuels that can deliver energy security while reducing environmental and health impacts without requiring disruptive changes to existing energy systems.

**Fig. 1:**
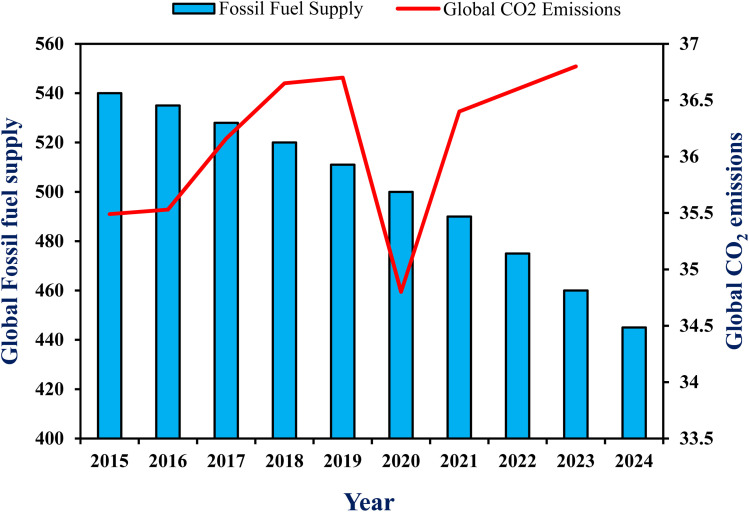
Global trends in fossil fuel supply and energy-related CO_2_ emissions over the past decade. The figure illustrates the continuing dependence of the global energy system on fossil resources despite increasing efforts toward decarbonization. Data were compiled and synthesized from recent reports published by the International Energy Agency (IEA), Energy Institute Statistical Review of World Energy, and the Global Carbon Project. The figure is intended to provide contextual background for the growing need for low-carbon liquid-fuel alternatives such as biodiesel.

The environmental implications of continued fossil fuel consumption further intensify this urgency. Persistent emissions of carbon dioxide and other greenhouse gases have already exceeded levels consistent with internationally agreed climate targets, accelerating global temperature rise and amplifying the frequency and severity of extreme climatic events.^5,6^ In parallel, fossil fuel combustion releases harmful air pollutants, including nitrogen oxides, sulfur oxides, and fine particulate matter, which are directly associated with respiratory and cardiovascular diseases.^7^ As illustrated in (Fig. 1), recent decade-scale trends show that even when fossil fuel supply experiences temporary reductions, global CO_2_ emissions rapidly recover and continue to increase. This divergence demonstrates that modest supply-side constraints alone are insufficient to achieve sustained emissions mitigation and underscores the necessity for fundamentally low-carbon liquid fuel alternatives.

Within this context, biodiesel has emerged as a strategically important renewable liquid fuel capable of addressing both environmental and infrastructural challenges. Biodiesel consists of fatty acid alkyl esters derived from biological lipids and exhibits favorable properties such as negligible sulfur content, enhanced biodegradability, high flash point, and reduced toxicity relative to petroleum diesel. Importantly, biodiesel can be utilized as a drop-in fuel or blended with conventional diesel in existing engines and distribution networks, thereby avoiding the need for extensive infrastructural modifications. Numerous life-cycle assessments have demonstrated that biodiesel can achieve substantial reductions in greenhouse gas emissions, particularly when produced from non-edible or waste-derived feedstocks. These characteristics position biodiesel as a technically feasible and immediately deployable option for lowering the carbon intensity of transport and industrial energy systems.^8–11^

The overall sustainability of biodiesel, however, is strongly dependent on feedstock selection. First-generation biodiesel derived from edible vegetable oils has raised concerns related to land-use change, food security, and limited scalability. These limitations motivated the development of second-generation biodiesel pathways based on waste oils, animal fats, and non-edible biomass, which offer improved environmental performance but introduce technical challenges associated with high free fatty acid and moisture contents. More recently, third-generation feedstocks, particularly microalgae, have attracted increasing attention due to their exceptional lipid productivity, rapid growth rates, ability to utilize non-arable land, and potential for direct carbon dioxide sequestration.^12–15^ The growing volume of scientific publications focused on microalgal biodiesel reflects a clear shift toward more resource-efficient and environmentally responsible feedstock systems.

Despite considerable progress, large-scale biodiesel production remains constrained by catalytic limitations, energy-intensive downstream processing, and economic competitiveness with fossil diesel. Conventional homogeneous catalysts offer high reaction rates but are highly sensitive to feedstock impurities and generate large volumes of wastewater.^16–18^ Consequently, research efforts have increasingly focused on heterogeneous and enzymatic catalysts, supercritical transesterification, advanced reactor configurations, and reaction enhancement techniques such as microwave and ultrasonic assistance.^19,20^ In parallel, the integration of biodiesel production within biorefinery frameworks—where fuels are co-produced alongside high-value chemicals and materials—has emerged as a key strategy for improving techno-economic feasibility and overall process sustainability.^21,22^

Against this background, the present review provides a comprehensive and critically integrated assessment of next-generation biodiesel production technologies, extending beyond conventional reviews that focus on individual aspects of feedstocks, catalysts, or conversion processes. Particular emphasis is placed on the evolution from first- and second-generation feedstocks to microalgal biorefinery platforms, the mechanistic foundations of homogeneous, heterogeneous, bifunctional, and enzymatic catalysis, and the growing contribution of process-intensification technologies to improved conversion performance and commercial implementation. The review further incorporates quantitative comparisons of catalytic performance, feedstock sustainability, operational stability, and technology readiness, while critically evaluating recent advances in reactor engineering, downstream processing, techno-economic analysis (TEA), life-cycle assessment (LCA), and commercialization challenges. By integrating catalytic science, process engineering, sustainability assessment, and commercialization perspectives within a unified framework, this review provides a holistic perspective and strategic roadmap for translating laboratory-scale advances into commercially competitive, low-carbon biodiesel technologies capable of supporting future energy-transition goals.

## 2. Biodiesel: fundamentals, properties, and sustainability metrics

Biodiesel is a renewable liquid fuel chemically defined as a mixture of mono-alkyl esters of long-chain fatty acids, commonly referred to as fatty acid alkyl esters (FAAEs). These compounds are typically produced through the transesterification of triglycerides derived from biological lipids with short-chain alcohols, most commonly methanol, yielding fatty acid methyl esters (FAME) and glycerol as a co-product. The defining molecular feature of biodiesel is the presence of ester functional groups, which introduce oxygen into the fuel structure and fundamentally distinguish biodiesel from petroleum diesel, which is composed almost entirely of hydrocarbons. This oxygenated nature governs key combustion characteristics, including ignition quality, flame propagation, and soot formation, and underpins many of the environmental and performance advantages associated with biodiesel use.^23–25^

To ensure engine compatibility, operational safety, and commercial acceptance, biodiesel must comply with internationally recognised fuel quality standards. The most widely adopted specifications are ASTM D6751 and EN 14214, which define allowable limits for critical physicochemical properties such as ester content, density, kinematic viscosity, flash point, sulfur concentration, acid value, water content, and oxidation stability. These parameters collectively govern fuel handling, storage stability, injection behaviour, and long-term engine durability.^26–28^ A comparative overview of these key fuel properties, together with representative performance and sustainability metrics for biodiesel, biodiesel blends, and petroleum diesel, is provided in Table 1. Compliance with such standards has been essential in enabling biodiesel to function as a drop-in or blend fuel within existing diesel infrastructure without requiring engine modification.

**Table 1:** Comparative physicochemical properties, fuel quality standards, and sustainability metrics of biodiesel and petroleum diesel.^38,39^

Parameter	Petroleum diesel	Biodiesel (B100)	Biodiesel blends (B5–B20)	Scientific significance/implication
Primary chemical composition	Hydrocarbons (alkanes, cycloalkanes, aromatics)	Fatty acid methyl esters (FAME)	Hydrocarbon–FAME mixture	Determines combustion chemistry, emissions, and lubricity
Oxygen content (wt%)	∼0	10–12	0.5–2.5	Oxygenated structure enhances combustion completeness
Sulfur content (ppm)	<10 (ULSD)	∼0	Proportional to blend ratio	Eliminates SO_2_ emissions and catalyst poisoning
Cetane number	40–55	50–65	45–58	Higher cetane improves ignition quality and reduces engine noise
Kinematic viscosity (40 °C, mm^2^ s^−1^)	2.0–4.5	4.0–6.0	2.5–4.8	Affects fuel injection, atomization, and spray pattern
Density (15 °C, kg m^−3^)	820–845	860–900	830–860	Influences volumetric fuel consumption
Flash point (°C)	55–75	>120	70–100	Higher flash point enhances storage and transport safety
Lower heating value (MJ kg^−1^)	42–45	37–40	40–43	Biodiesel has slightly lower energy density
Lubricity (HFRR, µm)	450–520	<300	<400	Biodiesel significantly improves injector wear protection
Cold flow properties (CFPP, °C)	−15 to −5	−5 to +10 (feedstock-dependent)	−10 to 0	Limitation for cold climates; mitigated by blending/additives
Oxidation stability (h, EN 14112)	High	Moderate (feedstock-dependent)	Improved *vs.* B100	Influenced by degree of unsaturation
Biodegradability (%)	<30 (28 days)	>90 (28 days)	>60	Reduces long-term environmental persistence
Toxicity	Moderate	Low/non-toxic	Low	Important for spill risk assessment
Life-cycle GHG emissions (g CO_2_-eq per MJ)	85–95	20–50	60–80	Strongly dependent on feedstock and processing route
ASTM D6751 compliance	Not applicable	Required	Required (blend limits apply)	Ensures fuel quality and engine compatibility
EN 14214 compliance	Not applicable	Required	Required (≤B7 in EU)	More stringent control of ester purity and iodine value

Global biodiesel production has expanded steadily over the past two decades, driven by renewable energy mandates, climate mitigation strategies, and concerns over energy security. Early large-scale deployment was concentrated in regions with strong agricultural capacity and supportive regulatory frameworks, particularly the European Union. More recently, biodiesel production has diversified geographically, with rapid growth observed in Southeast Asia, South America, and North America, reflecting increased utilisation of waste-derived and non-edible feedstocks. This expansion has been accompanied by a gradual transition away from first-generation edible oils toward second-generation and advanced feedstocks, motivated by sustainability criteria related to land use, carbon intensity, and resource efficiency. Despite these advances, biodiesel still represents a relatively small fraction of total global liquid fuel consumption, highlighting the need for continued technological innovation and feedstock diversification.^29–31^

From a sustainability perspective, biodiesel offers clear advantages over fossil diesel when assessed using life-cycle analysis frameworks. Carbon dioxide emissions generated during biodiesel combustion are partially offset by carbon uptake during biomass growth, resulting in significantly lower net greenhouse gas emissions compared with petroleum-derived fuels. The magnitude of this benefit depends strongly on feedstock origin, cultivation practices, and conversion pathways, with waste-based and advanced feedstocks consistently delivering the most favorable carbon balances. In addition to climate mitigation, biodiesel combustion typically produces lower emissions of carbon monoxide, unburned hydrocarbons, and particulate matter due to its oxygenated molecular structure and negligible aromatic content. These characteristics contribute to improved air quality and reduced public health risks, reinforcing biodiesel's role as a transitional low-carbon fuel for emission-intensive transport sectors.^32–34^

Beyond its environmental benefits, biodiesel exhibits favourable safety, handling, and engine performance characteristics. Biodiesel has a substantially higher flash point than petroleum diesel, reducing risks associated with storage, transport, and accidental ignition. It is also readily biodegradable and exhibits low toxicity, limiting long-term environmental damage in the event of fuel spills. From an engine performance perspective, biodiesel provides superior lubricity, which can significantly reduce wear in fuel injection systems, particularly in ultra-low sulfur diesel formulations. While challenges such as cold-flow limitations and oxidative instability may arise for certain feedstocks, these issues are increasingly addressed through blending strategies, additive technologies, and targeted feedstock selection.^35–37^ Collectively, these properties demonstrate that biodiesel is a technically mature and environmentally compatible fuel within existing diesel-based energy systems.

## 3. Biodiesel feedstocks: evolution from 1G to 3G systems

Biodiesel feedstocks are systematically classified into successive generations to reflect the progressive resolution of sustainability, scalability, and resource-efficiency constraints inherent to lipid-based fuel production. Early biodiesel development focused on lipid sources that were readily available and compatible with established conversion technologies; however, subsequent systems-level analyses have demonstrated that feedstock choice exerts a dominant influence on environmental impact, expansion potential, and long-term viability. While first- and second-generation feedstocks contribute to energy diversification and waste valorisation, they remain fundamentally constrained by agricultural land availability, feedstock volume, and competition for critical resources. In contrast, third-generation feedstocks represent a structural transition toward biologically intensive systems capable of decoupling fuel production from conventional agriculture.^40–42^ The key characteristics, limitations, and long-term deployment potential of each feedstock generation are comparatively summarized in Table 2, highlighting why feedstock evolution is central to future biodiesel development.

**Table 2:** Comparative analysis of biodiesel feedstock generations highlighting resource requirements, sustainability constraints, and scalability potential

Feedstock generation	Representative feedstocks	Typical lipid productivity	Land & water dependency	Key sustainability advantages	Major structural limitations	Scalability potential	Role in long-term biodiesel strategy	Ref.
First-generation (1G)	Soybean, rapeseed, palm, sunflower oils	Low–moderate (≈0.4–1.5 t oil ha^−1^ year^−1^)	High dependence on arable land and freshwater	Mature supply chains; uniform oil quality; simple processing	Food–fuel competition; indirect land-use change; biodiversity loss; yield ceiling	Low	Transitional pathway; unsuitable for long-term biodiesel expansion	57
Second-generation (2G)	Waste cooking oil, animal fats, grease trap residues	Variable, feedstock-limited	No additional land; indirect water footprint	Waste valorisation; reduced life-cycle emissions; avoids food competition	High FFA and moisture; heterogeneous composition; limited and dispersed availability	Moderate (volume-limited)	Supplementary feedstock; improves sustainability but cannot meet global demand alone	58
Third-generation (3G)	Microalgae (chlorella, nannochloropsis, scenedesmus), cyanobacteria	Very high (up to 10–30 t oil ha^−1^ year^−1^, system-dependent)	No arable land; saline water, wastewater, effluents	Exceptional areal productivity; land independence; CO_2_ capture; nutrient recycling	Cultivation, harvesting, and downstream processing costs	High (theoretical)	Promising long-term platform for future biodiesel production	59
Emerging/hybrid systems	Oleaginous yeasts, fungi, integrated waste-to-lipid platforms	High (reactor-based; substrate-dependent)	Minimal land; depends on carbon substrate	Flexible feedstocks; circular bioeconomy integration	Early technological readiness; scale-up uncertainty	Moderate–high (conditional)	Complementary platforms that may enhance feedstock diversification and integrated biorefinery development	60

First-generation biodiesel feedstocks are derived from edible vegetable oils, including soybean, rapeseed, palm, and sunflower oils. Their initial adoption was driven by mature agricultural supply chains, predictable lipid composition, and straightforward compatibility with conventional transesterification processes. Nevertheless, large-scale reliance on edible oils introduces intrinsic sustainability constraints that limit their long-term applicability. The diversion of food-grade lipids to fuel production intensifies food–fuel competition and contributes to indirect land-use change, freshwater depletion, and biodiversity loss. Furthermore, the intrinsic yield limitations of oilseed crops impose strict ceilings on areal lipid productivity, preventing meaningful scale-up without substantial environmental trade-offs. Consequently, first-generation feedstocks are increasingly recognized as transitional resources that enabled early biodiesel market entry but cannot serve as a long-term foundation for future biofuel expansion.^43–45^

Second-generation biodiesel feedstocks encompass waste-derived and non-edible lipid resources such as waste cooking oils, animal fats, and industrial grease residues. These feedstocks offer clear environmental benefits by valorising waste streams, reducing disposal-related emissions, and avoiding direct competition with food production. However, their utilisation introduces significant technical and logistical challenges. Elevated free fatty acid and moisture contents, together with chemical heterogeneity and impurities, complicate conversion processes and often require additional pretreatment steps. In parallel, the geographically dispersed and finite nature of waste lipid streams restricts their capacity to support large-scale, centralised biodiesel production. While second-generation feedstocks substantially improve sustainability metrics relative to edible oils, their limited availability constrains their role to that of a complementary resource rather than a long-term globally deployable solution.^46–48^

Third-generation biodiesel feedstocks, particularly microalgae and cyanobacteria, overcome the fundamental limitations associated with terrestrial lipid sources. These photosynthetic microorganisms exhibit rapid growth rates, high photosynthetic efficiencies, and exceptional areal lipid productivities. Crucially, microalgae can be cultivated on non-arable land using saline water, wastewater, or industrial effluents, effectively eliminating competition with food production and freshwater resources. Their capacity to directly assimilate carbon dioxide enables biodiesel production to be integrated with carbon capture, nutrient recycling, and environmental remediation strategies.^15,49–51^ Collectively, these attributes position microalgae among the most promising feedstock classes for addressing land, water, carbon, and scalability challenges associated with biodiesel production. Nevertheless, their widespread commercial deployment remains dependent on continued advances in cultivation technologies, harvesting efficiency, and overall process economics.

Beyond classical generational categories, emerging feedstock concepts aim to further intensify lipid production through biological and process integration. Oleaginous yeasts and fungi have demonstrated the capacity to accumulate lipids from diverse carbon substrates under controlled fermentation conditions, offering flexibility in feedstock utilization.^52,53^ In parallel, integrated waste-to-lipid platforms combine wastewater treatment, nutrient recovery, and microbial lipid biosynthesis, enhancing overall resource efficiency within circular bioeconomy frameworks. Despite their promise, these approaches remain constrained by scale-up challenges and feedstock logistics. Importantly, many hybrid systems increasingly incorporate microalgae as central components, reinforcing rather than displacing their pivotal role. As such, emerging concepts should be viewed as complementary strategies that strengthen, rather than replace, the central position of microalgae in next-generation biodiesel feedstock architectures.^54–56^

From a critical sustainability and deployment perspective, no single feedstock generation currently satisfies all technical, economic, and environmental requirements for large-scale biodiesel production. First-generation feedstocks benefit from mature supply chains and established conversion technologies but remain constrained by food–fuel competition and land-use pressures. Second-generation feedstocks offer improved environmental performance through waste valorisation; however, their finite and geographically dispersed availability limits long-term scalability. Third-generation microalgal systems provide unparalleled areal lipid productivity, carbon capture capability, and independence from arable land, making them among the most promising long-term biodiesel feedstocks. Nevertheless, their commercial implementation remains hindered by high cultivation, harvesting, dewatering, and lipid extraction costs, which continue to challenge economic competitiveness with conventional and waste-derived lipid sources. Consequently, future biodiesel deployment is likely to rely on integrated and feedstock-diversified strategies that balance environmental sustainability, resource efficiency, technological maturity, and economic feasibility rather than depending exclusively on a single feedstock platform.^61,62^

## 4. Biodiesel production technologies: from conventional to advanced

Biodiesel production technologies have evolved in response to the chemical nature of lipid feedstocks, fuel quality requirements, and industrial scalability constraints. Broadly, these technologies can be classified into non-transesterification routes and transesterification-based routes. While multiple pathways have been explored historically, only a subset has demonstrated compatibility with fuel standards, engine requirements, and large-scale deployment. Importantly, the selection of production technology is not independent of feedstock characteristics, but rather tightly coupled to lipid composition, impurity tolerance, and downstream separation requirements. As feedstock complexity increases from refined edible oils to waste-derived and microalgal lipids, process robustness and controllability become decisive factors.^63,64^ This section therefore provides a critical overview of biodiesel production routes, positioning transesterification as the dominant industrial pathway and establishing the rationale for a catalyst-centred discussion in the subsequent section.

Several non-transesterification approaches have been investigated as alternatives to conventional biodiesel synthesis, including direct blending of vegetable oils with diesel, microemulsion formation, and thermochemical conversion such as pyrolysis. These routes were primarily explored to simplify processing and avoid chemical conversion steps. However, practical deployment has been limited by intrinsic fuel and operational constraints. Direct blending suffers from high viscosity, poor atomisation, and long-term engine durability issues. Microemulsions improve flow properties but exhibit phase instability and inconsistent combustion behaviour. Pyrolysis can convert lipids into hydrocarbon-like fuels, yet requires high energy input and produces complex product distributions that often fail to meet diesel fuel specifications without extensive upgrading. Consequently, these approaches remain structurally constrained and largely confined to laboratory or niche applications, rather than forming viable industrial biodiesel pathways.^65–67^

Transesterification has emerged as the dominant and globally standardised route for biodiesel production due to its chemical efficiency, feedstock flexibility, and compliance with international fuel specifications. In this process, triglycerides react with short-chain alcohols to form fatty acid alkyl esters and glycerol. The reaction proceeds under comparatively mild conditions and can accommodate a wide range of lipid sources, from refined vegetable oils to waste-derived and microalgal lipids, provided appropriate process control is applied. Crucially, transesterification yields products that meet ASTM and EN biodiesel standards when properly optimised, enabling direct integration into existing diesel infrastructure. Unlike alternative routes, transesterification offers predictable reaction pathways, high conversion efficiencies, and well-established reactor configurations, establishing it not as one option among many, but as the only production technology that has demonstrated sustained industrial viability across diverse feedstocks.^68–70^

A defining feature of transesterification is the formation of glycerol as a stoichiometric by-product, which introduces additional separation and purification requirements. Phase behaviour, reaction completeness, and product quality are highly sensitive to feedstock composition, particularly free fatty acid and water content. These sensitivities directly influence downstream processing complexity, waste generation, and overall process economics. As a result, biodiesel production efficiency is not governed solely by reaction chemistry, but by the ability to control side reactions, phase separation, and impurity tolerance under realistic feedstock conditions. These challenges are fundamentally catalyst-dependent, with catalyst performance determining conversion efficiency, selectivity, and operational robustness.^71–73^ Consequently, the transition from production routes to catalyst design is not incremental but essential, necessitating a focused examination of catalytic systems, which is addressed in the following section.

## 5. Catalytic systems for biodiesel production

### 5.1. Catalyst selection criteria

The selection of catalytic systems for biodiesel production must be governed by the physicochemical realities of practical feedstocks rather than idealised laboratory conditions. Waste-derived oils and microalgal lipids typically contain elevated free fatty acids (FFAs), residual moisture, suspended solids, and dissolved impurities, all of which directly influence catalyst performance and stability. Among these factors, FFA tolerance is critical, as FFAs readily undergo saponification in base-catalysed systems, leading to emulsion formation, yield loss, and difficult downstream separation. Acidic catalysts and bifunctional systems circumvent this limitation by promoting esterification alongside transesterification, while enzymatic catalysts intrinsically accommodate FFAs without side reactions. Recent systematic analyses emphasise that catalyst effectiveness must therefore be evaluated within a feedstock-dependent operational envelope rather than by intrinsic activity alone.^74,75^

Beyond FFA content, water sensitivity, catalyst recovery, and durability under repeated or continuous operation play a central role in determining the economic and operational feasibility of biodiesel production processes. Residual moisture—particularly prevalent in microalgal and waste lipid streams—suppresses the activity of conventional base catalysts and accelerates deactivation through hydrolysis and dilution effects. In contrast, solid acid catalysts and lipase-based systems retain activity in moderately wet environments, enabling simplified pretreatment and reduced energy input for drying. At the process level, homogeneous catalysts impose high separation and wastewater treatment costs, whereas heterogeneous catalysts and immobilised enzymes facilitate recovery and reuse but may suffer from leaching, fouling, or gradual deactivation.^76,77^ Collectively, these interrelated factors highlight the necessity of feedstock-specific catalyst selection and optimization strategies. To facilitate a critical and data-driven comparison, Table 3 quantitatively evaluates the principal catalytic systems employed in biodiesel production with respect to catalytic performance, operational stability, feedstock compatibility, process limitations, and industrial readiness.

**Table 3:** Quantitative comparison of catalytic systems for biodiesel production, highlighting catalytic performance, feedstock tolerance, operational stability, and industrial deployment potential^a^

Catalyst class	Representative catalysts	Typical optimized biodiesel/FAME yield (%)	Operational stability/reusability	FFA tolerance & water sensitivity	Typical feedstock compatibility	Key process advantages	Intrinsic limitations & penalties	Industrial readiness (TRL)	Ref.
Homogeneous alkaline catalysts	NaOH, KOH, NaOCH_3_, KOCH_3_	95–99	Non-recyclable	Low FFA tolerance (<1 wt%); highly sensitive to moisture	Refined vegetable oils and low-FFA lipids	Very fast kinetics, high conversion efficiency, low catalyst cost, mild operating conditions	Soap formation, emulsion generation, catalyst loss, extensive washing and wastewater production	High (commercially established)	131
Homogeneous acid catalysts	H_2_SO_4_, HCl, *p*-toluenesulfonic acid	85–98	Non-recyclable	High FFA tolerance (>5–20 wt%); moderate water tolerance	Waste cooking oils, animal fats, acidic algal lipids, high-FFA feedstocks	Simultaneous esterification and transesterification, suitable for poor-quality feedstocks	Slow reaction kinetics, corrosive environment, high alcohol consumption, increased energy demand	High (primarily pretreatment applications)	132
Heterogeneous basic catalysts	CaO, MgO, hydrotalcites, mixed metal oxides, biomass-derived ashes	90–99	Stable for approximately 5–10 reuse cycles under optimized conditions	Low–moderate FFA tolerance (<2–3 wt%); sensitive to moisture and CO_2_	Refined oils, conditioned waste oils, low-FFA microalgal oils	Easy catalyst recovery, catalyst reuse, reduced wastewater generation, suitability for continuous processing	Catalyst leaching, carbonation, active-site poisoning, deactivation by water and CO_2_	Moderate–high	133
Heterogeneous solid acid catalysts	Sulfonated carbons, amberlyst resins, SBA-15 derivatives, sulfated metal oxides, heteropoly acids	80–96	Typically 5–15 reuse cycles, depending on acid-site stability	High FFA tolerance (>10 wt%); moderate water tolerance	Waste oils, sludge lipids, residual algal lipids, wet feedstocks	Simultaneous esterification and transesterification, no soap formation, simplified catalyst separation	Lower intrinsic activity, longer reaction times, acid-site leaching, diffusional limitations	Moderate	134
Bifunctional heterogeneous catalysts	Acid–base composites, supported mixed oxides, hierarchical multifunctional catalysts	90–98	Typically 5–10 reuse cycles	Moderate–high FFA tolerance	Mixed triglyceride/FFA feedstocks and low-quality oils	One-pot esterification and transesterification, reduced pretreatment requirements, process simplification	Active-site neutralization, synthesis complexity, diffusional resistance, scale-up challenges	Emerging–moderate	135
Enzymatic catalysis	Free and immobilized lipases (*e.g.*, novozym 435)	85–99	Frequently 10–50 operational cycles for immobilized systems	Very high tolerance toward FFAs and water	Highly heterogeneous, wet, acidic, and waste-derived lipid feedstocks	High-purity biodiesel, mild operating conditions, excellent feedstock flexibility, minimal side reactions	High enzyme cost, alcohol inhibition, slower reaction rates, mass-transfer limitations	Moderate (specialized and high-value applications)	136
Intensified catalytic systems	Microwave-, ultrasound-, and cavitation-assisted transesterification	Typically provide 10–80% reduction in reaction time and/or 5–30% enhancement in conversion relative to conventional systems	Catalyst dependent	Catalyst dependent	Broad range of feedstocks, including low-quality oils and biomass-derived lipids	Enhanced heat and mass transfer, accelerated reaction kinetics, reduced residence time, improved process efficiency	Specialized equipment requirements, energy-distribution challenges, scale-up complexity	Moderate–high potential	137

a Values represent representative performance ranges reported under optimized operating conditions in the cited literature and are intended for comparative evaluation of catalyst classes. Actual performance may vary depending on catalyst composition, feedstock characteristics, reactor configuration, alcohol-to-oil ratio, and process conditions.

### 5.2. Fundamental reaction mechanisms in biodiesel transesterification

The synthesis of biodiesel through transesterification and esterification reactions is fundamentally governed by molecular-level catalytic interactions involving triglycerides, free fatty acids (FFAs), alcohol molecules, and catalytically active sites. Independent of catalyst type, biodiesel formation proceeds *via* activation of the acyl carbon within lipid substrates followed by nucleophilic substitution pathways that yield fatty acid alkyl esters (FAAEs) and glycerol-derived intermediates. The efficiency of these transformations is strongly influenced by catalyst electronic structure, surface acidity/basicity, adsorption–desorption equilibria, transition-state stabilisation, and interfacial mass-transfer phenomena occurring within multiphase reaction systems. Recent advances integrating density functional theory (DFT), *operando* spectroscopy, surface characterisation, and kinetic modelling have significantly expanded mechanistic understanding of catalyst–substrate interactions, revealing the critical roles of oxygen vacancies, active-site cooperativity, and catalyst microenvironment in governing catalytic activity and stability. Simultaneously, mechanistic elucidation of catalyst deactivation pathways—including coke deposition, alcohol inhibition, active-site poisoning, and structural destabilisation—has become essential for improving long-term process durability and scalability in advanced biodiesel and integrated microalgal biorefinery systems.^68,78^

#### 5.2.1. Base-catalysed transesterification mechanism

Homogeneous alkaline catalysis remains the most widely implemented industrial route for biodiesel production owing to its exceptionally rapid reaction kinetics and high conversion efficiencies under comparatively mild operating conditions. In alkaline systems, catalysts such as NaOH, KOH, and sodium methoxide react with short-chain alcohols to generate highly reactive alkoxide ions, primarily methoxide (CH_3_O^−^), which function as the principal nucleophilic species responsible for triglyceride conversion. The catalytic mechanism is initiated by nucleophilic attack of methoxide ions on the electrophilic carbonyl carbon of triglyceride ester bonds, generating unstable tetrahedral intermediates that subsequently collapse to release fatty acid methyl esters (FAMEs) and partially converted glyceride species. The overall reaction proceeds through consecutive and reversible stages involving triglyceride, diglyceride, and monoglyceride intermediates prior to final glycerol formation. Owing to the strong nucleophilicity of alkoxide ions and the relatively low activation energy associated with alkaline catalysis, conversion efficiencies frequently exceed 95% within short reaction times under optimised conditions.^79,80^

Despite its kinetic advantages, alkaline transesterification remains intrinsically sensitive to feedstock impurities, particularly free fatty acids and residual water. FFAs readily react with alkaline catalysts through saponification reactions, producing soaps that consume active catalytic species, increase emulsion stability, and significantly complicate downstream phase separation and glycerol purification. Simultaneously, water promotes hydrolysis of triglycerides, increasing free fatty acid concentration and accelerating secondary soap–formation pathways. Recent mechanistic and reactor-engineering studies demonstrate that observed reaction kinetics in alkaline transesterification are often governed not solely by intrinsic catalytic chemistry but also by interfacial mass-transfer limitations arising from immiscible oil–alcohol phases. Consequently, intensified mixing systems, cavitation-assisted reactors, co-solvent integration, and microstructured reactor designs are increasingly employed to minimise diffusion resistance and improve interfacial contact efficiency.^81,82^

#### 5.2.2. Acid-catalysed esterification and transesterification mechanisms

Acid-catalysed biodiesel synthesis proceeds through proton-mediated activation pathways fundamentally distinct from alkoxide-driven alkaline transesterification and is particularly advantageous for high-free-fatty-acid feedstocks. In Brønsted-acid systems, strong proton donors such as sulfuric acid protonate the carbonyl oxygen atom of triglycerides and free fatty acids, thereby increasing electrophilicity of the adjacent acyl carbon and facilitating nucleophilic attack by alcohol molecules. Following protonation, methanol attacks the activated carbonyl centre, generating protonated tetrahedral intermediates that subsequently undergo proton transfer, bond rearrangement, and elimination reactions to yield fatty acid methyl esters and water or glyceride intermediates. Unlike alkaline systems, acid catalysis enables simultaneous esterification of FFAs and transesterification of triglycerides within a single reaction environment, thereby eliminating soap formation and enabling direct processing of acidic waste oils and low-quality lipid streams without extensive pretreatment.^83,84^

Despite its superior tolerance toward FFAs and moisture, acid catalysis exhibits intrinsically slower kinetics due to the mechanistic nature of proton-mediated activation. The reaction pathway depends on reversible protonation equilibria and comparatively weaker nucleophilic activation of alcohol molecules, resulting in higher activation energies and lower overall reaction rates relative to alkaline systems. Consequently, acid-catalysed biodiesel production generally requires elevated alcohol-to-oil molar ratios, prolonged reaction times, and higher operating temperatures to achieve satisfactory conversion efficiencies. Furthermore, water generated during esterification can shift reaction equilibrium toward reactants and suppress catalytic activity unless continuously removed from the reaction medium. Recent studies indicate that catalyst acidity, hydrophobicity, and water-management capability critically influence catalytic stability and long-term process efficiency, motivating increasing integration of microwave heating, ultrasound irradiation, and reactive-separation strategies within intensified acidic transesterification systems.^85,86^

#### 5.2.3. Surface-mediated heterogeneous catalytic mechanisms

Heterogeneous biodiesel catalysis is governed by surface-mediated physicochemical interactions occurring at catalytically active solid interfaces, where adsorption, activation, surface reaction, and product desorption collectively determine catalytic performance. Unlike homogeneous catalytic systems, heterogeneous catalysts operate through accessible acidic or basic sites distributed across porous solid matrices, rendering catalytic efficiency highly dependent on surface morphology, pore architecture, active-site accessibility, and transport dynamics. In heterogeneous basic catalysts, including CaO-based systems and mixed metal oxides, surface O^2−^ species facilitate alcohol deprotonation and generate surface-bound alkoxide intermediates capable of nucleophilic attack on triglyceride carbonyl groups. Simultaneously, Lewis basic centres enhance carbonyl polarisation and increase susceptibility to nucleophilic substitution. In heterogeneous acid systems, Brønsted acid sites protonate carbonyl oxygen atoms, whereas Lewis acid centres coordinate electron-rich oxygen atoms and promote electron-density redistribution during catalytic activation.^87,88^

Recent mechanistic investigations increasingly emphasise the decisive role of oxygen vacancies, coordinatively unsaturated metal centres, and metal–oxygen interfacial interactions in governing catalytic activity, selectivity, and stability. Oxygen-deficient sites improve adsorption of methanol and triglycerides while facilitating transition-state stabilisation and charge redistribution during transesterification reactions. In bifunctional catalysts, coexistence of acidic and basic active sites enables simultaneous esterification and transesterification, thereby improving feedstock flexibility and simplifying process flowsheets. Nevertheless, heterogeneous catalytic systems remain susceptible to diffusion limitations, pore blockage, coke deposition, catalyst leaching, and active-site deactivation during prolonged operation. Consequently, recent catalyst-development strategies increasingly focus on hierarchical pore engineering, hydrophobic surface modification, nanostructured active phases, and stabilised multifunctional interfaces capable of simultaneously improving catalytic accessibility, mass-transfer efficiency, and long-term operational durability.^86,89^

#### 5.2.4. Enzymatic transesterification mechanism

Enzymatic biodiesel production, predominantly catalysed by lipases, proceeds through highly selective acyl-transfer mechanisms fundamentally distinct from conventional chemical catalysis. Lipases contain a catalytic triad generally composed of serine, histidine, and aspartate (or glutamate) residues that cooperatively mediate ester-bond cleavage and reformation within the enzyme active site. During transesterification, the nucleophilic hydroxyl group of the active-site serine attacks the triglyceride carbonyl carbon, producing a transient acyl–enzyme intermediate and releasing partially converted glyceride species. Subsequent reaction of alcohol molecules with the acyl–enzyme complex regenerates the active enzyme while simultaneously producing fatty acid alkyl esters. This cyclic catalytic pathway enables simultaneous esterification and transesterification without soap formation, even in the presence of elevated water and free fatty acid concentrations, thereby rendering lipase-mediated systems highly compatible with heterogeneous and low-quality lipid feedstocks.^90,91^

A defining mechanistic feature of lipases is interfacial activation, whereby catalytic activity is significantly enhanced at oil–water or oil–alcohol interfaces owing to conformational rearrangement of enzyme structure and exposure of hydrophobic active-site domains. Lipases additionally exhibit substrate specificity toward particular fatty acid chain lengths, stereochemical configurations, and degrees of unsaturation, thereby influencing biodiesel composition and product selectivity. Despite these mechanistic advantages, enzymatic systems remain vulnerable to alcohol-induced inhibition, conformational destabilisation, and gradual loss of catalytic activity during repeated operation. Excess methanol concentrations may disrupt enzyme tertiary structure, interfere with hydration layers surrounding the biocatalyst, and reduce catalytic turnover efficiency. Consequently, recent enzymatic biodiesel systems increasingly employ immobilisation strategies, controlled alcohol feeding, nanostructured support materials, and metal–organic-framework-based biocatalytic platforms to preserve enzyme conformation and improve operational stability during intensified biodiesel production processes.^92,93^

### 5.3. Homogeneous catalysis

Homogeneous alkaline catalysis (KOH, NaOH, sodium alkoxides) derives its industrial attractiveness from fast methoxide-mediated nucleophilic attack on triglyceride carbonyls, producing rapid conversion to FAME under mild temperatures and modest alcohol excess. The reaction pathway is well established: base deprotonates alcohol → alkoxide nucleophile → tetrahedral intermediate → methyl ester + glycerolate. Because kinetics are largely mass-transfer limited in immiscible oil–alcohol systems, reactor configuration and mixing (*e.g.*, intensified, microstructured reactors) strongly influence observed space–time yields and selectivity. In refined, low-FFA, dry oils, base catalysis routinely achieves >95% conversion within minutes, making it the benchmark route for established plants and pilot demonstrations.^94–96^ Despite kinetic advantages, alkali systems are fundamentally feedstock-fragile. FFAs react with alkali to form soaps (saponification), which stabilise emulsions, increase interfacial viscosity, complicate phase disengagement, and escalate washwater volumes and disposal costs. Residual moisture has similar deleterious effects *via* hydrolysis and catalyst dilution. Therefore, homogeneous base processes for heterogeneous or wet lipids require rigorous feedstock conditioning (drying, acid esterification, blending with low-FFA oil) or process intensification (microreactor hydrodynamics, co-solvent strategies) to remain viable at scale. The engineering trade-off is clear: exceptional kinetics *versus* increased upstream costs and downstream separation complexity.^97,98^

Recent studies demonstrate that the practical limitations of homogeneous base catalysis for complex lipid feedstocks can be alleviated through reactor intensification and feedstock engineering. Tsaoulidis *et al.* (2024) showed that KOH-catalysed transesterification of waste cooking oil in a 1 mm cross-flow microreactor achieved >90% FAME within seconds to minutes by optimising hydrodynamics, residence time, and reactant ratios, establishing mass-transfer enhancement as a decisive factor rather than intrinsic reaction kinetics.^99^ Complementarily, Jain *et al.* (2025) demonstrated that blending microalgal oil with a small fraction of waste cooking oil enables single-step NaOH-catalysed transesterification at mild temperature (50 °C), yielding ∼92% FAME without acid pretreatment.^100^ Together, these studies indicate that homogeneous base catalysis, while intrinsically feedstock-fragile, can be extended to heterogeneous and high-FFA lipid systems through intelligent reactor design and strategic feedstock modification.

Homogeneous acid catalysis (principally H_2_SO_4_) provides direct processing capability for high-FFA feedstocks because Brønsted acids catalyse esterification of free fatty acids to methyl esters and can also promote triglyceride transesterification (albeit more slowly). Mechanistically, protonation of carbonyl oxygen increases electrophilicity of the acyl carbon, enabling nucleophilic methanol attack and water elimination. Acid routes avoid saponification and thus permit one-pot treatment of acidic oils; they are therefore frequently employed as a first-stage pretreatment (esterification) to reduce FFA prior to base transesterification or, in cases of very high acidity, as the principal catalyst system.^101,102^ The drawbacks are kinetic and materials penalties: acid-catalysed conversions require higher alcohol ratios, elevated temperatures, longer reaction times, and corrosion-resistant equipment; neutralisation of spent acid and salt by-products increases downstream processing burdens. To mitigate these drawbacks, researchers have developed process-intensification strategies (co-solvents, ultrasound, low-temperature optimisation, and continuous removal of water) that accelerate esterification, lower energy demand, and shrink reaction volumes. When paired with efficient intensification, homogeneous acid routes remain a practical pathway for low-cost, high-FFA lipids until robust heterogeneous or enzymatic alternatives reach comparable TRLs.^103,104^

Recent advances in acid-catalysed pretreatment strategies highlight process intensification as a viable route to manage high FFA feedstocks with reduced energy and reagent demands. Saeed *et al.* (2023) employed central composite design to optimise H_2_SO_4_-catalysed esterification, demonstrating that judicious use of co-solvents and water-adsorption/entrainer removal effectively enhances FFA conversion at lower temperatures and acid loadings, offering practical operational recipes that minimise energy input and corrosive reagent usage.^105^ In parallel, Thawornprasert *et al.* (2025) combined high-intensity ultrasound with Amberlyst-15 catalysis and a preliminary soaking step to esterify palm fatty acid distillate (PFAD), achieving >89% methyl-ester purity and showing catalyst recyclability over multiple cycles.^106^ Together, these studies illustrate that integration of intensification techniques with acid catalysis can substantially improve the tractability of high-FFA lipid streams, bridging the performance gap between conventional homogeneous acid systems and more robust heterogeneous processes.

Although homogeneous catalytic systems exhibit intrinsically rapid reaction kinetics, the overall efficiency of biodiesel transesterification is frequently governed by interfacial mass-transfer limitations arising from the immiscibility of oil and alcohol phases. Consequently, effective reactor hydrodynamics and mixing behaviour become decisive factors controlling conversion efficiency, reaction selectivity, and space–time yield. Recent kinetic investigations demonstrate that intensified reactor configurations—including microreactors, oscillatory-flow systems, cavitation-assisted reactors, and continuous-flow devices—substantially enhance interfacial contact area, reduce diffusion resistance, and accelerate methanol–triglyceride interactions under both alkaline and acidic catalytic conditions. In particular, microstructured reactors operating under laminar yet highly controlled flow regimes have shown exceptional capability for shortening reaction times from hours to seconds while maintaining high FAME yields and improved thermal management. These improvements originate from enhanced phase dispersion, rapid heat transfer, and minimisation of concentration gradients within confined reaction channels, thereby shifting the overall process from diffusion-controlled toward kinetically controlled regimes.^69,107^

Beyond reactor engineering, non-conventional process-intensification strategies such as ultrasound irradiation, microwave-assisted heating, co-solvent integration, and reactive water-removal systems have emerged as highly effective approaches for overcoming kinetic and thermodynamic limitations associated with homogeneous catalysis. Ultrasonic cavitation enhances transesterification rates through formation and collapse of microbubbles that generate intense localised turbulence, interfacial disruption, and transient high-energy microenvironments, thereby improving reactant miscibility and accelerating catalytic conversion. Similarly, microwave-assisted systems promote rapid volumetric heating and selective dipolar polarisation of polar reactants, leading to accelerated molecular collision frequency and reduced reaction times. In acid-catalysed systems, continuous water removal and co-solvent-assisted phase homogenisation are particularly important for shifting equilibrium toward ester formation and suppressing reverse hydrolysis reactions. Collectively, these intensified processing strategies significantly reduce energy demand, catalyst loading, and residence time while improving the industrial feasibility of homogeneous catalytic biodiesel production for complex and high-free-fatty-acid feedstocks.^95,108^

### 5.4. Heterogeneous catalysis

Heterogeneous basic catalysts, including CaO-based materials, mixed metal oxides, and hydrotalcite-derived solids, have attracted sustained interest for biodiesel production owing to their facile separation, reusability, and reduced wastewater generation relative to homogeneous alkaline systems. Their catalytic activity originates from surface basic sites that promote alcohol deprotonation and subsequent nucleophilic attack on triglyceride carbonyl groups, enabling efficient transesterification under suitable conditions. However, large-scale deployment remains constrained by intrinsic stability issues, most notably alkaline leaching of Ca^2+^ species in methanolic media, surface carbonation from atmospheric CO_2_, and sintering during repeated reaction cycles, which progressively diminish catalytic performance. Consequently, recent research has shifted from maximising initial activity toward improving structural durability and resistance to deactivation through dopant incorporation, lattice stabilisation, and multifunctional catalyst design.^109,110^ For example, Yeneneh and Sufe (2025) demonstrated that Ni-doped eggshell-derived CaO catalysts achieved ∼98% FAME yield with improved reusability by enhancing crystallinity and suppressing calcium leaching during transesterification.^111^ Similarly, Foroutan *et al.* (2026) reported that modified CaO catalysts integrated with magnetic and perovskite components delivered high biodiesel yields (≈97–99%) and robust recyclability, confirming that catalyst stabilisation strategies are decisive for extending the industrial relevance of heterogeneous base-catalysed biodiesel systems.^112^

For high-FFA lipid feedstocks, heterogeneous solid acid catalysts—most notably sulfonated carbons, supported heteropoly acids, and acidic polymeric resins—provide a critical advantage by enabling simultaneous esterification of free fatty acids and transesterification of triglycerides without soap formation. Their catalytic performance is primarily dictated by the density and accessibility of Brønsted acid sites, surface hydrophobicity to maintain activity in methanol-rich media, and resistance to coke deposition and sulfur leaching under prolonged operation. In this context, biomass-derived sulfonated carbons have attracted increasing attention due to their circular-economy compatibility, tunable acidity, and low material cost; however, maintaining acid site stability during cyclic or continuous operation remains a central challenge. Recent studies therefore focus on controlled sulfonation chemistry, hierarchical pore architectures, and robust carbon backbones to enhance durability.^113,114^ For example, Yang *et al.* (2024) demonstrated that sulfonated biochar catalysts derived from *Camellia oleifera* shell biomass enabled efficient biodiesel production, achieving >90% yield with good recyclability, thereby confirming that controlled sulfonation and hydrophobic carbon frameworks are critical for maintaining Brønsted acid site stability during repeated esterification–transesterification cycles.^115^ Similarly, Kumar (2024) reported that date-seed-derived sulfonated carbon catalysts exhibited high free-fatty-acid conversion efficiency and enhanced water tolerance, highlighting the decisive role of surface hydrophobicity and carbon backbone robustness in suppressing deactivation and enabling durable heterogeneous acid catalysis for high-FFA lipid feedstocks.^116^

Supported and bifunctional heterogeneous catalysts are increasingly explored as an integrated solution for one-pot processing of complex lipid feeds by combining acidic and basic functionalities within a single solid matrix. Such systems—typically based on metal oxides dispersed on porous supports or bifunctional composites—are designed to esterify free fatty acids while simultaneously transesterifying triglycerides, thereby simplifying process flowsheets. However, effective catalyst design requires careful control of acid–base site proximity and strength to prevent mutual neutralisation, pore blockage, and diffusional limitations for bulky triglyceride molecules. Recent studies show that pore architecture, active-phase dispersion, and anchoring stability are more decisive for sustained performance than nominal acidity or basicity, particularly under continuous operation.^110,117^ For example, Tariq *et al.* (2025) developed bifunctional alkaline-earth-metal-oxide catalysts supported on WO_3_@MCM-41, achieving ∼93–96% biodiesel yields from waste and refined oils *via* simultaneous esterification–transesterification, while highlighting the importance of balanced acid–base site distribution.^98^ Similarly, Liu *et al.* (2025) developed a zinc–barium/montmorillonite acid–base bifunctional catalyst for acidified palm oil, attaining a maximum biodiesel yield of 96.1% with strong resistance to deactivation and enhanced water tolerance, thereby underscoring the critical role of accessible active sites and robust pore architectures for industrially viable supported catalytic systems.^118^

The catalytic efficiency of heterogeneous biodiesel systems is fundamentally governed by the physicochemical nature, accessibility, and electronic environment of surface active sites, which collectively regulate adsorption, activation, and transformation of lipid substrates. In heterogeneous basic catalysts, surface oxygen anions (O^2−^), hydroxyl groups, and coordinatively unsaturated metal–oxygen pairs function as strong basic centres capable of deprotonating alcohol molecules and generating surface-bound alkoxide intermediates that subsequently attack triglyceride carbonyl groups. Conversely, heterogeneous acid catalysts predominantly operate through Brønsted acid sites, which protonate carbonyl oxygen atoms and increase electrophilicity of acyl carbons, or Lewis acid sites, which coordinate electron-rich oxygen atoms and induce carbonyl polarisation through electron-density redistribution. Recent mechanistic investigations further demonstrate that catalyst–substrate interactions are strongly influenced by active-site density, surface hydroxyl concentration, metal–support electronic interactions, and oxygen-vacancy distribution, all of which directly affect methanol activation, triglyceride adsorption, transition-state stabilisation, and desorption of fatty acid methyl esters (FAMEs) and glycerol products. Consequently, rational engineering of active-site architecture has become central to the development of highly efficient heterogeneous biodiesel catalysts capable of processing structurally complex and impurity-rich lipid feedstocks.^114,119^

The long-term operational stability of heterogeneous biodiesel catalysts is strongly governed by structure–activity relationships involving surface area, pore architecture, hydrophobicity, and active-site distribution. Hierarchical mesoporous frameworks and interconnected pore networks facilitate diffusion of bulky triglyceride molecules toward catalytically accessible interfaces while minimising internal mass-transfer limitations. Simultaneously, hydrophobic surface properties improve catalyst durability in methanol-rich and water-containing systems by suppressing competitive adsorption of polar species and reducing hydrolytic deactivation. Nevertheless, heterogeneous catalysts remain susceptible to progressive deactivation through alkaline leaching, coke deposition, surface carbonation, sulfur loss, sintering, and active-site poisoning by strongly adsorbed intermediates or impurities. Consequently, recent catalyst-engineering strategies increasingly emphasise lattice stabilisation, nanostructured active phases, robust metal–support interactions, and electronic modulation of catalytic interfaces to enhance resistance against structural degradation during cyclic and continuous operation. These findings demonstrate that heterogeneous biodiesel catalysis is governed not solely by catalyst composition but by dynamic coupling between surface chemistry, transport phenomena, and catalyst microstructure under realistic reaction environments.^117^

### 5.5. Enzymatic catalysis

Enzymatic biodiesel production, primarily catalysed by lipases, represents a mechanistically robust route for converting complex and high-free-fatty-acid lipid feedstocks that are incompatible with conventional alkaline systems. Lipases intrinsically catalyse both esterification of free fatty acids and transesterification of triglycerides without soap formation and retain catalytic activity in the presence of water, thereby enabling operation under mild temperatures and atmospheric pressure. These features lead to the formation of high-purity fatty acid methyl esters and glycerol with minimal side reactions, substantially reducing downstream separation and purification demands. Nevertheless, enzymatic processes are characterised by slower intrinsic kinetics relative to chemical catalysis, and enzyme deactivation—particularly in the presence of excess short-chain alcohols—remains a critical limitation. As a result, enzymatic routes are best suited to applications where feedstock heterogeneity, product quality, and low-temperature operation outweigh the need for maximum throughput.^69,120^ In this context, Alonazi *et al.* (2023) demonstrated that combined immobilised bacterial lipases supported on CaCO_3_ enabled solvent-free biodiesel production from spent coffee grounds oil, achieving >99% conversion under optimised conditions, thereby confirming the practical viability of low-cost immobilised enzymatic systems for waste-derived lipid feedstocks.^121^

Immobilisation is a pivotal strategy for enhancing the industrial feasibility of enzymatic biodiesel production, as it improves enzyme structural stability, enables efficient recovery and reuse, and moderates alcohol-induced deactivation by creating controlled microenvironments around the biocatalyst. Immobilised lipases supported on inorganic matrices, polymeric carriers, or hybrid materials generally exhibit prolonged operational lifetimes and enhanced tolerance to methanol, particularly when coupled with stepwise alcohol addition or the use of tailored reaction media. Despite these advantages, high enzyme cost and gradual loss of catalytic activity over successive cycles remain significant economic constraints that limit large-scale deployment. Consequently, current research increasingly emphasises rational carrier design, co-immobilisation approaches, and interfacial stabilisation strategies aimed at increasing effective turnover numbers and reducing catalyst replacement frequency.^122,123^ In this context, Mangiagalli *et al.* (2024) systematically demonstrated that short-chain alcohols induce conformational destabilisation of the commercial lipase Novozym 435, providing direct evidence that controlled methanol dosing and appropriate solvent selection are essential to preserve enzyme structure and catalytic activity during prolonged transesterification processes.^124^

**Fig. 2:**
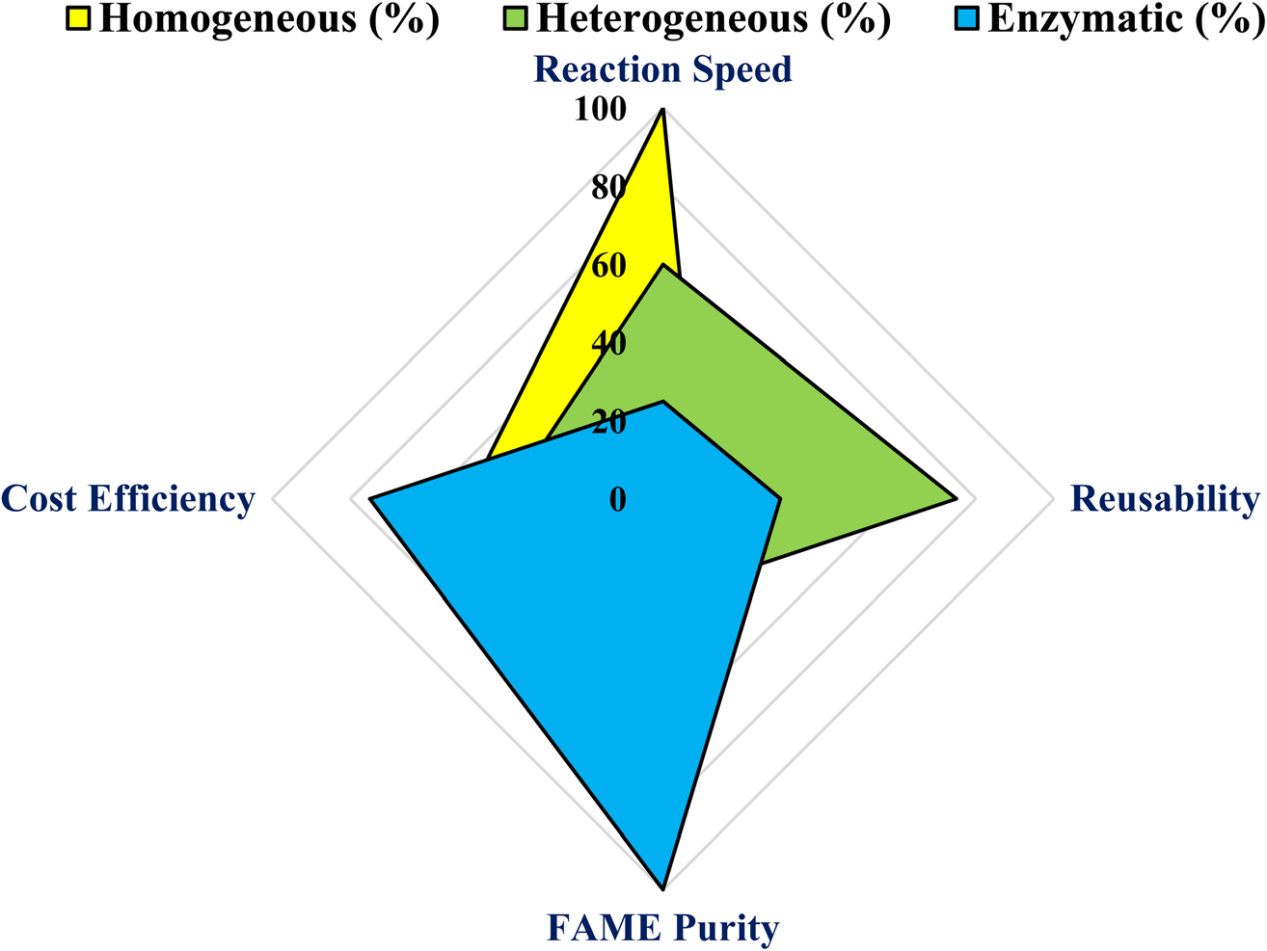
Comparative assessment of homogeneous, heterogeneous, and enzymatic catalytic systems for biodiesel production, illustrating relative trade-offs among reaction kinetics, reusability, FAME purity, and cost efficiency. Comparative scores were derived from normalized literature-based performance indicators summarized in Table 3, including catalytic activity, operational stability, product quality, and economic characteristics. The figure is intended to provide a qualitative-to-semiquantitative comparison of catalyst classes rather than absolute performance values.

Process-intensification strategies are increasingly employed to overcome the inherently slow kinetics of enzymatic biodiesel production and to narrow the performance gap relative to conventional chemical catalysis. Techniques such as ultrasound-assisted transesterification, optimised reaction media, and tailored reactor configurations are designed to enhance mass-transfer efficiency and interfacial contact while preserving enzyme structural integrity. These approaches are particularly effective when combined with immobilised enzymes and controlled alcohol dosing, as they simultaneously improve reaction rates and operational stability. Although large-scale industrial deployment remains limited, recent studies demonstrate that intensified enzymatic routes can be competitive for processing low-quality, high-FFA lipid feedstocks within integrated biorefinery concepts.^125,126^ For example, Moschona *et al.* (2024) optimised enzymatic transesterification of acid oil using a low-cost Biolipasa-R lipase, achieving high FAME yields under mild conditions and demonstrating that systematic process optimisation and enzyme reuse can substantially improve enzymatic viability.^127^ Similarly, Liow *et al.* (2025) showed that ultrasound-assisted enzymatic transesterification, augmented by a CO_2_-based alkyl carbamate additive, significantly enhanced reaction rates and biodiesel yields while mitigating enzyme inhibition, underscoring the effectiveness of non-thermal intensification strategies in advancing enzymatic biodiesel production.^128^ Collectively, the performance of enzymatic catalysis must be interpreted within the broader context of catalytic system trade-offs, where superior product quality and feedstock flexibility are achieved at the expense of reaction rate and economic scalability. When compared with homogeneous and heterogeneous catalytic systems, enzymatic routes occupy a distinct operational niche characterised by high selectivity and environmental compatibility but constrained by kinetic and cost limitations, as summarised in (Fig. 2).

### 5.6. Critical evaluation and industrial perspective of catalytic systems

Despite substantial advances in catalyst development, no single catalytic system currently satisfies all requirements for high conversion efficiency, feedstock flexibility, operational stability, environmental sustainability, and economic viability simultaneously. Homogeneous alkaline catalysts remain the dominant industrial technology owing to their rapid reaction kinetics, high fatty acid methyl ester (FAME) yields, and technological maturity. However, their sensitivity to free fatty acids and water, coupled with intensive downstream purification and wastewater generation, restricts their applicability to low-quality feedstocks. In contrast, heterogeneous catalysts offer significant advantages in catalyst recovery, recyclability, and process sustainability, making them attractive candidates for continuous biodiesel production. Nevertheless, catalyst leaching, active-site deactivation, pore blockage, and long-term stability remain major barriers to widespread commercial implementation. Enzymatic catalysts provide superior selectivity, excellent tolerance toward heterogeneous feedstocks, and environmentally benign processing conditions; however, high enzyme costs, limited operational lifetimes, and slower reaction kinetics continue to hinder large-scale deployment. Consequently, future biodiesel production is unlikely to rely on a universal catalytic platform. Rather, catalyst selection will increasingly depend on feedstock characteristics, process configuration, and techno-economic constraints. Recent studies further suggest that the most promising pathway may involve integrating robust heterogeneous or immobilised enzymatic catalysts with intensified reactor technologies to simultaneously enhance catalytic performance, process efficiency, and industrial scalability while reducing overall environmental impact.^95,129,130^

## 6. Advanced biodiesel production technologies and process intensification

Conventional catalytic transesterification remains the industrial benchmark for biodiesel production; however, its practical applicability is fundamentally constrained by high free fatty acid (FFA) content, residual moisture, and multiphase mass-transfer limitations inherent to oil–alcohol systems. These constraints reduce reaction efficiency, promote side reactions such as saponification, and significantly increase downstream purification and wastewater treatment demands when low-quality feedstocks, including waste oils and biomass-derived lipids, are employed. Consequently, recent research has shifted toward advanced biodiesel production technologies that re-engineer reaction environments through intensified transport phenomena, integrated reaction–separation, and continuous processing architectures. Within this context, process intensification has emerged as a unifying strategy that synergistically combines reactor engineering, energy integration, and novel facilitation techniques to overcome equilibrium limitations, expand feedstock tolerance, and improve scalability and energy efficiency beyond the capabilities of conventional batch reactors. Importantly, these intensified platforms do not displace catalytic mechanisms but extend their operational envelope into regimes that are critical for cost-effective, low-carbon biodiesel production from heterogeneous and water-rich feedstocks.^9,138,139^

### 6.1. Supercritical alcohol transesterification

Supercritical alcohol transesterification is a non-catalytic biodiesel production route in which triglycerides react with short-chain alcohols under temperatures and pressures exceeding the alcohol's critical point, generating a homogeneous single-phase reaction medium. In this state, alcohols such as methanol or ethanol exhibit enhanced solvent power and gas-like diffusivity, leading to complete miscibility with non-polar lipids and elimination of interfacial mass-transfer resistance. This physicochemical transformation enables direct conversion of triglycerides and free fatty acids into fatty acid alkyl esters without the need for acidic, basic, or enzymatic catalysts, thereby avoiding saponification and catalyst deactivation phenomena that limit conventional transesterification processes.^140,141^

Despite its exceptional tolerance to high free fatty acid and moisture contents, supercritical transesterification is associated with demanding operating conditions, typically requiring temperatures above 240 °C and pressures in the range of 8–15 MPa. These conditions impose significant energy penalties and necessitate advanced reactor materials, pressure-resistant equipment, and rigorous safety protocols. Recent system-level evaluations indicate that the overall efficiency of supercritical routes depends critically on heat recovery, energy integration, and continuous operation strategies, without which their energy intensity can exceed that of optimised catalytic processes. Consequently, supercritical alcohol transesterification is most appropriately deployed within integrated biorefinery configurations that can exploit shared high-temperature utilities or for feedstocks whose quality renders catalytic pathways impractical.^142,143^

Recent experimental work has provided updated evidence for the efficacy and optimisation of supercritical transesterification under industrially relevant conditions. Ceran *et al.* (2025) investigated biodiesel synthesis from *Jatropha curcas* oil under supercritical methanol conditions using a ZnO/γ-Al_2_O_3_ catalyst, demonstrating that optimized alcohol-to-oil molar ratios, elevated temperatures (∼300 °C), and 9 MPa pressure can yield ∼95% fatty acid methyl esters (FAME) in residence times as short as minutes, highlighting both kinetic advantages and catalyst tolerance at supercritical states.^144^ Complementing this, Lin *et al.* (2024) assessed the application of supercritical methanol transesterification to high-acid oil feedstocks, reporting successful conversion of low-quality oils to biodiesel with competitive fuel properties and no pretreatment requirement, thereby underscoring the robustness of supercritical alcohol systems for heterogeneous feedstocks.^145^ Together, these studies confirm that, while demanding in terms of temperature and pressure, supercritical methanol transesterification can achieve high conversions and feedstock flexibility when supported by appropriate reactor design and process optimisation.

### 6.2. Reactor engineering for intensified biodiesel production

Reactor architecture fundamentally determines how intrinsic transesterification kinetics translate into practical conversion efficiency, selectivity, and industrial productivity. Traditional batch reactors suffer from limited oil–alcohol interfacial contact, inefficient heat transfer, and broadly distributed residence times, which constrain effective mass transfer and reduce yield. To overcome these limitations, research has shifted toward intensified reactors that restructure flow dynamics, enhance interfacial surface area, and integrate reaction–separation phenomena, enabling improved process efficiency and scalability. Recent work highlights the critical importance of such reactor innovations for continuous biodiesel synthesis, showing that reactor design improvements can significantly boost conversion and reduce energy consumption compared to conventional systems.^146,147^

#### 6.2.1. Continuous‐flow and microstructured reactors

Continuous-flow and microstructured reactors have been shown to dramatically enhance biodiesel production by increasing surface-to-volume ratios and shortening diffusion paths, leading to improved mixing, heat transfer, and reduced mass-transfer resistance. In a recent experimental study, a helical tubular reactor coupled with a static mixer achieved biodiesel conversion increases up to ∼91% within 8 min by optimising residence time and catalyst concentration, demonstrating enhanced mass and heat transfer relative to conventional continuous systems.^148^ Similarly, emerging work on 3D-printed static mixers integrated into continuous microreactors shows that tailored internal geometries can further improve mixing efficiency and conversion when processing crude palm oil feedstocks, highlighting the potential of additive manufacturing to enhance reactor performance and facilitate industrial implementation.^149^ Despite these advances, microstructured systems still face engineering challenges including fouling control, pressure drop management, and long-term stability under continuous operation.

#### 6.2.2. Reactive distillation and integrated reaction–separation systems

Reactive distillation merges chemical conversion and *in situ* separation within a single unit, enabling simultaneous transesterification and product removal that shifts equilibrium toward biodiesel formation and reduces alcohol excess requirements. Recent process sustainability assessments demonstrate that reactive distillation configurations outperform conventional reactor–distillation systems in both energy utilisation and environmental impact, with lower specific energy consumption and improved conversion for homogeneous and heterogeneous base catalysts.^150^ Although many published simulations focus on integrated designs rather than experimental trials, these findings confirm that combining reaction and separation can reduce unit operations and enhance process intensification when lipid streams are favourable for vapour–liquid equilibrium control.

#### 6.2.3. *In Situ* and direct transesterification approaches

*In situ* and direct transesterification integrate lipid extraction and biodiesel formation by converting lipids directly within biomass matrices, eliminating discrete drying and solvent recovery steps that dominate energy use in biomass-derived feedstocks. Recent advances in process design show that *in situ* approaches can yield competitive fatty acid alkyl ester productivity from wet feedstocks when catalysts and reactor conditions are tuned to tolerate moisture and cellular components, potentially lowering capital and operating costs.^151^ However, integrating these reactions in continuous or intensified reactors requires catalysts that remain stable in complex matrices and downstream purification strategies that can handle residual proteins and pigments. Therefore, reactor engineering that enhances solvent penetration and internal mass transfer remains a key research priority for practical *in situ* biodiesel systems.^152^

### 6.3. Reaction enhancement and non-thermal intensification techniques

Non-thermal intensification techniques such as microwave and ultrasonic irradiation have been extensively investigated as means to accelerate biodiesel transesterification without necessitating proportionate increases in bulk reaction temperatures. Microwave irradiation enhances volumetric heating and promotes strong dipolar interactions between methanol and triglyceride molecules, which can reduce reaction time and improve conversion efficiency.^153,154^ For example, Chavez-Esquivel *et al.* (2025) applied microwave-assisted transesterification using a sustainable eggshell-derived SrFe/CaO heterogeneous catalyst and reported ∼98.9% FAME yield under microwave conditions with optimized power and methanol/oil ratio, demonstrating both high efficiency and process intensification *via* electromagnetic energy input.^155^ These results illustrate how microwave energy can improve heat and mass transport within the reacting mixture, enabling high conversion even with low-cost catalysts and reducing the effective activation energy for the transesterification reaction.

Ultrasonic irradiation intensification leverages acoustic cavitation to generate localized high temperature and pressure microzones, disrupt phase boundaries, and dramatically enhance micro-mixing between immiscible reactants.^156^ In a recent study by Li *et al.* (2024), ultrasound-assisted transesterification of waste coconut scum oil using a CoFe_2_O_4_@sulfonated graphene oxide (SGO) magnetic nanocatalyst yielded ∼96.9% biodiesel under mild conditions, while maintaining catalyst stability over multiple reuse cycles, highlighting the capacity of ultrasonic cavitation to improve both kinetics and catalyst performance.^157^ Despite these promising findings, challenges remain regarding energy efficiency, uniform field distribution, and reactor design for scale-up, making the integration of non-thermal techniques with continuous or hybrid reactors an active field of research toward economically viable large-scale biodiesel production.

### 6.4. Critical evaluation and industrial perspective of process intensification technologies

Despite the substantial performance improvements reported for supercritical processing, microstructured reactors, reactive distillation, and non-thermal intensification techniques, their industrial adoption remains considerably more challenging than laboratory-scale studies often suggest. Although these technologies can significantly enhance reaction kinetics, mass-transfer efficiency, and biodiesel yield, their practical implementation is frequently constrained by increased capital investment, process complexity, equipment durability requirements, and scale-up limitations. For example, supercritical transesterification eliminates catalyst-related challenges and enables direct processing of low-quality feedstocks; however, its high energy demand and severe operating conditions may offset economic and environmental benefits unless supported by effective heat-integration strategies. Similarly, microreactors and intensified flow systems demonstrate exceptional conversion efficiencies but face throughput, fouling, and process-control challenges during large-scale operation. Microwave- and ultrasound-assisted systems accelerate reaction rates; nevertheless, achieving uniform energy distribution and maintaining energy efficiency at industrial scales remain significant engineering barriers. Consequently, the future success of process intensification technologies will depend not solely on conversion performance but on their ability to deliver favourable techno-economic outcomes, operational reliability, and seamless integration within commercial biodiesel and biorefinery infrastructures.^158–160^

## 7. Microalgal biodiesel systems: integrated carbon-to-lipid conversion and process engineering framework

**Fig. 3:**
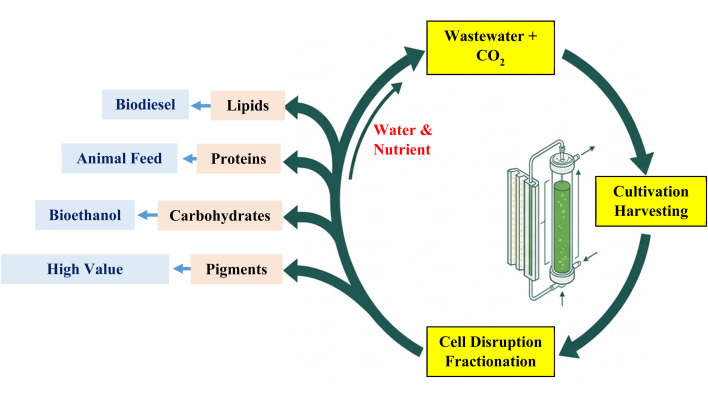
Integrated microalgal biorefinery illustrating CO_2_ and wastewater utilisation, biomass conversion, and fractionation into biodiesel and value-added products within a circular system.

Microalgal biodiesel systems represent a highly promising third-generation biofuel platform capable of enabling low-carbon liquid-fuel production without direct competition for arable land, freshwater resources, or food supply chains. In contrast to first- and second-generation feedstocks, microalgae exhibit superior photosynthetic efficiency, rapid biomass accumulation, and an enhanced capacity for lipid biosynthesis under controlled cultivation conditions. These attributes result in lipid productivities that substantially exceed those of conventional terrestrial oil crops on an areal basis, while cultivation can be implemented on non-arable land using saline water, industrial effluents, or municipal wastewater streams. Importantly, microalgae directly assimilate carbon dioxide during growth, facilitating the integration of biodiesel production with carbon capture, nutrient recovery, and environmental remediation strategies. Within this context, microalgal biodiesel production is increasingly conceptualised as an integrated biorefinery system, in which carbon inputs, cultivation processes, biomass conversion, and multi-product valorisation are systematically interconnected, as illustrated in (Fig. 3). However, the large-scale deployment of microalgal biodiesel remains dependent on the coordinated optimisation of cultivation systems, downstream processing, and catalytic conversion pathways, as highlighted by recent experimental and process-intensified studies summarised in Table 4.

**Table 4:** Recent catalytic and process-intensified strategies for biodiesel production from microalgal and algae-derived lipid feedstocks

Catalyst system class	Feedstock/microalgae type	Catalyst composition & structural features	Dominant catalytic/mechanistic function	Process configuration & intensification strategy	Representative performance window (headline)	Key scientific significance & scalability implication	Ref.
Carbon-based carbocatalyst (graphene oxide)	*Chlorella vulgaris* (wet, *in situ*)	Oxygen-rich graphene oxide sheets; high surface acidity; microwave-responsive	Acid-catalysed *in situ* esterification–transesterification *via* surface –COOH/–OH groups	Pulsed microwave-assisted *in situ* methanolysis	≈ 96% FAME under optimised microwave conditions	Demonstrates feasibility of single-step conversion of wet biomass with reduced downstream energy demand	203
Heterogeneous basic oxide (CaO/hydrotalcite)	*Nannochloropsis* sp.	CaO dispersed on layered double hydroxide; strong surface basicity	Base-catalysed transesterification of extracted lipids	Batch *in situ* process with co-solvent (*n*-hexane)	Moderate–high crude biodiesel yield (condition-dependent)	Highlights mass-transfer limitations and pretreatment needs for basic catalysts in algal systems	204
Magnetic sulfonated carbon (solid acid)	Lipid-extracted algal residue/wet paste	Sulfonated carbon matrix impregnated with Fe_3_O_4_; magnetic separability	Brønsted-acid-driven esterification and transesterification	Microwave-assisted one-step conversion of *wet* biomass	High conversion reported	Enables catalyst recovery and processing of residual algal biomass streams	205
Magnetic alkaline nanocatalyst (NaOH/CoFe_2_O_4_)	Waste cooking oil (transferable to algal oils)	NaOH-functionalised CoFe_2_O_4_ nanoparticles; high dispersion; magnetic recovery	Strong base-catalysed transesterification	Conventional heating with magnetic separation	≈ 98.7% FAME (WCO benchmark)	Demonstrates catalyst potency and recyclability applicable to algal oil analogues	206
Calcium methoxide on magnetic biochar	*Chlorella* sp. (cultivated algal oil)	Eggshell-derived Ca(OCH_3_)_2_ anchored on magnetised biochar	Highly active methoxide-mediated transesterification	High methanol-to-oil ratio; magnetic recovery	High FAME conversion reported	Combines waste-derived catalyst synthesis with magnetic separability	207
Immobilised lipase on MOF (ZIF-8)	Wet microalgal biomass	Lipase immobilised within ZIF-8 pores; enhanced enzyme stability	Enzymatic esterification–transesterification without soap formation	Mild temperature; solvent-switchable system	Improved stability and conversion *vs.* free enzyme	Demonstrates biocatalytic robustness for high-FFA, wet algal feeds	208
Ultrasonic-assisted magnetic nanocatalyst (CoFe_2_O_4_@SGO)	Waste lipid feeds (transferable)	Core–shell CoFe_2_O_4_@SO_3_H-GO; high surface area	Acid-catalysed transesterification enhanced by cavitation	Ultrasound-assisted reactor; RSM optimisation	≈ 95–98% FAME	Demonstrates synergy between nanocatalysis and non-thermal intensification	157
Sulfonated biochar (solid acid)	Algal residues/residual biomass	Porous biochar functionalised with –SO_3_H groups	Brønsted acid esterification of FFAs	Microwave-assisted esterification	Effective FFA conversion	Highlights circular biochar catalysts for residue valorisation	209
Green sulfonated biochar catalyst	*Chlorella vulgaris*	Agricultural-waste-derived sulfonated carbon	Acid-catalysed esterification–transesterification	Conventional heating	Efficient catalytic activity	Demonstrates sustainable catalyst synthesis with competitive performance	115
MOF-based magnetic nanocatalyst (UiO-66-NH_2_@MnFe_2_O_4_)	Utilised edible oil (transferable to algal oils)	Amino-functional MOF grown on magnetic ferrite core	Coordinatively enhanced transesterification	Microwave-assisted reactor	High conversion with magnetic retrievability	Illustrates MOF robustness and catalyst recovery in biodiesel systems	210
Process & solvent-integrated systems	Wet algal biomass	Green solvents + biological preconditioning	Integrated lipid extraction and *in situ* transesterification	Wet conversion workflows	Competitive yields with reduced energy input	Addresses downstream bottleneck through process integration	211
Hydrodynamic cavitation-assisted catalysis	Algal oil simulants/WCO	Cavitation reactors with heterogeneous catalysts	Mass-transfer-driven rate enhancement	Continuous-flow cavitation	Marked reaction acceleration	Demonstrates scalability potential of cavitation-based intensification	212
Two-stage heterogeneous acid → base route	Wet microalgae slurries	Solid acid pre-esterification followed by base catalysis	Sequential FFA reduction and triglyceride conversion	Mild two-step process	High net FAME yield	Practical pathway for wet, high-FFA algal feeds	213

### 7.1. Systems-level sustainability and productivity framework of microalgal biodiesel

The sustainability rationale for microalgal biodiesel is rooted in its ability to decouple lipid production from the agricultural constraints that fundamentally limit terrestrial feedstocks. Microalgae can achieve lipid productivities one to two orders of magnitude higher than oilseed crops due to continuous cultivation, high carbon fixation efficiency, and short biomass turnover times. Unlike waste-derived lipid systems, which are inherently constrained by feedstock availability and saturation effects, microalgae offer theoretically expandable production capacity limited primarily by infrastructure and resource integration rather than biological supply. Furthermore, the use of non-potable water sources and direct utilisation of CO_2_-rich gas streams enables microalgal biodiesel to function as both a renewable fuel pathway and an environmental remediation strategy.^161^ Life-cycle assessments increasingly indicate that meaningful greenhouse gas mitigation is achievable only when such integration is implemented, reinforcing the necessity of system-level design rather than isolated process optimization.^162^

### 7.2. Carbon assimilation and trophic integration in microalgae

Microalgae utilise three principal trophic strategies-photoautotrophy, heterotrophy, and mixotrophy-which collectively determine carbon acquisition mechanisms, intracellular energy transduction, and the partitioning of metabolic fluxes toward biomass formation and lipid biosynthesis. These trophic modes are not merely physiological variations but represent distinct metabolic frameworks that govern carbon fixation efficiency, redox balance, and resource utilisation under different environmental and operational conditions. Photoautotrophy is the most extensively exploited mode, wherein microalgae capture solar radiation and assimilate atmospheric CO_2_ as the exclusive carbon source through oxygenic photosynthesis.^163–165^ Within the chloroplast, light-dependent reactions generate ATP and NADPH, which subsequently drive carbon fixation and biosynthetic pathways leading to carbohydrate, protein, and lipid formation. Despite its environmental sustainability and direct coupling to CO_2_ sequestration, photoautotrophic cultivation is intrinsically constrained by photon availability, light attenuation, and self-shading at high cell densities, as well as diurnal variations that impose intermittent productivity, thereby limiting achievable biomass concentrations and areal productivity.^166,167^

Heterotrophic growth represents an alternative metabolic strategy in which microalgae utilise reduced organic carbon substrates, such as glucose, acetate, or glycerol, as both carbon and energy sources in the absence of light.^168,169^ This trophic mode bypasses the limitations associated with light-dependent metabolism, enabling continuous cultivation, significantly higher cell densities, and enhanced volumetric productivities in controlled bioreactor systems. From a process engineering perspective, heterotrophic cultivation benefits from well-established fermentation technologies, improved process controllability, and reduced sensitivity to environmental fluctuations. Under optimised conditions, species including *Chlorella*, *Galdieria*, and *Crypthecodinium* exhibit elevated biomass accumulation and enhanced synthesis of storage lipids and high-value biochemicals.^170^ However, the requirement for external organic carbon introduces substantial economic and sustainability challenges, as substrate costs can dominate operational expenses and may compete with food and agricultural resources, thereby limiting large-scale feasibility.^165^

**Fig. 4:**
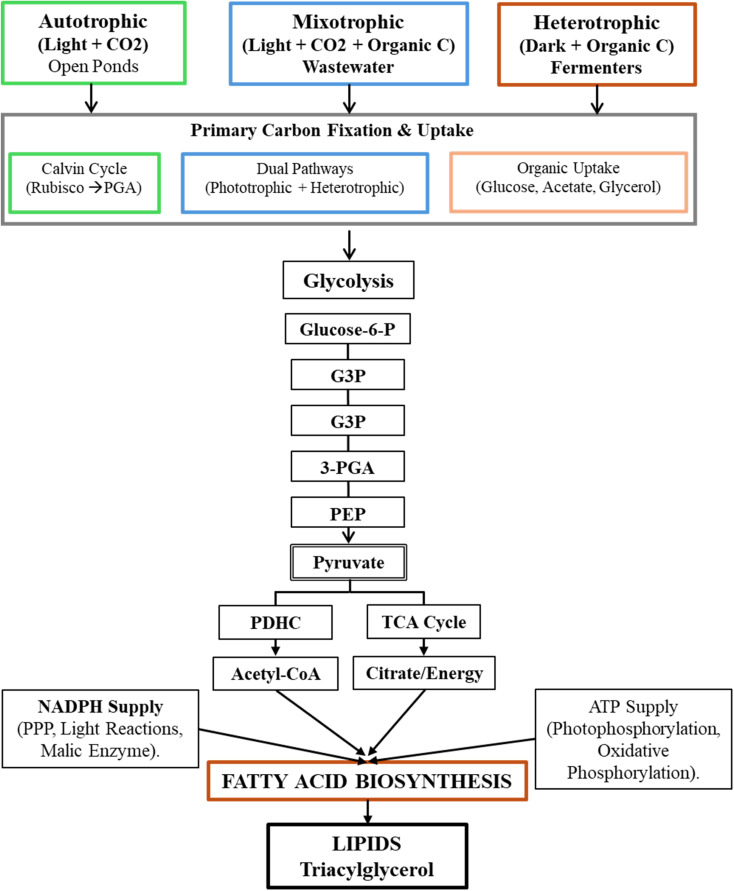
Integrated carbon assimilation and central metabolic pathways in microalgae under autotrophic, heterotrophic, and mixotrophic conditions, illustrating the convergence of carbon flux into pyruvate and acetyl-CoA for lipid biosynthesis.

Mixotrophy integrates both photoautotrophic and heterotrophic metabolic pathways, enabling microalgae to simultaneously assimilate inorganic carbon *via* photosynthesis and organic carbon through respiratory metabolism.^171^ In this dual-mode system, microalgae simultaneously perform photosynthesis while assimilating external carbon sources, resulting in synergistic metabolic interactions. Oxygen generated during photosynthesis supports respiratory processes, while CO_2_ released through respiration can be reassimilated, forming an internal carbon recycling loop that enhances overall carbon utilisation efficiency.^172^ Consequently, mixotrophic systems frequently exhibit biomass and lipid productivities that exceed the additive performance of purely photoautotrophic and heterotrophic cultures, a phenomenon described as “synergistic enhancement”.^172,173^ This enhanced performance arises from complementary metabolic pathways that optimise energy utilisation and reduce carbon loss. Additionally, mixotrophic cultivation typically requires lower concentrations of organic substrates compared with fully heterotrophic systems, thereby improving economic feasibility.^174,175^ Comparative analyses consistently indicate that both heterotrophic and mixotrophic modes outperform photoautotrophy in terms of biomass productivity and lipid accumulation, as demonstrated for *Chlorella vulgaris* and *Chlorella sorokiniana*.^176,177^ The convergence of these trophic pathways and their integration into central carbon metabolism, ultimately directing carbon flux toward lipid biosynthesis, is schematically illustrated in (Fig. 4).

Collectively, these trophic strategies define the fundamental carbon assimilation landscape of microalgal systems and exert a decisive influence on metabolic flux distribution, lipid biosynthesis efficiency, and overall process performance. The selection and optimisation of trophic mode are therefore not isolated biological considerations but central design parameters that must be aligned with reactor configuration, resource availability, and downstream conversion pathways. Understanding the interplay between carbon assimilation mechanisms and metabolic regulation provides a critical foundation for advancing microalgal biodiesel systems toward higher productivity, improved resource efficiency, and integrated biorefinery development.

### 7.3. Metabolic regulation and carbon flux toward lipid precursors

**Fig. 5:**
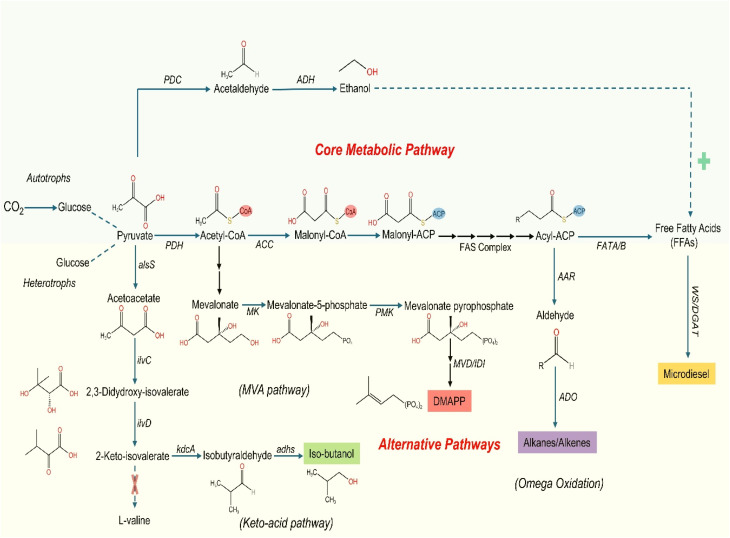
Integrated metabolic network illustrating carbon flux partitioning in microalgae from central carbon metabolism (pyruvate and acetyl-CoA) toward fatty acid biosynthesis and alternative biofuel pathways, including FAEE (biodiesel), alkanes, and isobutanol.

Fatty acid biosynthesis in microalgae is governed by a highly integrated metabolic network in which carbon flux is dynamically distributed among pathways supporting cellular energy generation, biomass formation, and lipid accumulation. Carbon assimilated under photoautotrophic, heterotrophic, and mixotrophic conditions ultimately converges at central metabolic intermediates, particularly pyruvate, which serves as a key metabolic junction linking upstream carbon assimilation with downstream lipid biosynthesis, as illustrated in (Fig. 5).^178,179^ The efficiency of carbon partitioning through this hub strongly influences the balance between growth-associated metabolism and storage lipid accumulation, thereby determining biodiesel precursor formation and overall process performance.

Pyruvate is converted into acetyl-CoA *via* the pyruvate dehydrogenase (PDH) complex, generating the principal precursor pool for fatty acid biosynthesis. Acetyl-CoA is subsequently converted into malonyl-CoA by acetyl-CoA carboxylase (ACC), the rate-limiting step of fatty acid synthesis.^180–182^ Through elongation reactions mediated by the Type II fatty acid synthase (FAS) system, malonyl-derived carbon is incorporated into long-chain acyl–ACP intermediates, predominantly within the C16–C18 range.^178,182,183^ These intermediates represent critical branching points that can either generate free fatty acids (FFAs) for lipid accumulation or be redirected toward hydrocarbon-producing pathways involving acyl–ACP reductase (AAR) and aldehyde deformylating oxygenase (ADO), forming the biochemical basis for advanced biofuel synthesis.^184,185^

Carbon allocation toward lipid biosynthesis is further influenced by competing metabolic pathways that function as carbon sinks or alternative product routes. Pyruvate may be diverted toward fermentative metabolism through pyruvate decarboxylase (PDC) and alcohol dehydrogenase (ADH), thereby reducing carbon availability for lipid synthesis. Alternatively, carbon can enter branched-chain amino acid pathways that generate intermediates for biofuel molecules such as isobutanol. In addition, FFAs derived from acyl–ACP may be converted into fatty acid ethyl esters (FAEEs) through the activity of wax ester synthase/acyl–CoA acyltransferase (WS/DGAT), providing a direct route toward biodiesel-like compounds. Consequently, metabolic engineering strategies increasingly focus on redirecting carbon flux away from competing pathways and toward lipid-derived fuel production.^183,185–187^

Collectively, the metabolic architecture governing fatty acid biosynthesis in microalgae is determined by the interaction between central carbon metabolism, regulatory enzymatic nodes, and competing flux pathways. Acetyl-CoA and acyl–ACP remain pivotal control points regulating carbon allocation between biomass formation and fuel-oriented products. Accordingly, metabolic engineering approaches increasingly target these nodes through enzyme overexpression, pathway repression, and transcriptional regulation to enhance lipid biosynthesis. Recent advances demonstrate that coordinated manipulation of these metabolic control points, together with process-level optimisation, can substantially improve lipid productivity and facilitate the efficient production of biodiesel and advanced biofuels in microalgal systems.

### 7.4. Microalgae strain selection and lipid accumulation strategies

Microalgae strain selection constitutes a foundational design decision in biodiesel production systems, as it simultaneously governs biomass productivity, lipid yield, biochemical composition, and operational robustness under large-scale conditions. Although numerous microalgal species are capable of accumulating substantial lipid fractions, particularly neutral lipids suitable for biodiesel synthesis, overall process viability is dictated by areal lipid productivity, which integrates growth rate, culture density, and lipid content. Consequently, strains that exhibit rapid cell division, high photosynthetic efficiency, and tolerance to fluctuating environmental conditions are prioritised over those achieving extreme lipid accumulation only under laboratory-controlled stress.^188,189^ Species such as *Chlorella*, *Scenedesmus*, and *Nannochloropsis* have therefore emerged as leading industrial candidates due to their adaptability to non-sterile outdoor cultivation, resistance to contamination, and compatibility with diverse water sources, including saline and wastewater streams. In contrast, slow-growing, high-lipid strains often suffer from low volumetric productivity, instability under open cultivation, and sensitivity to nutrient and light variability, which collectively undermine scalability. As a result, strain selection increasingly emphasises process resilience and sustained productivity, rather than maximising lipid percentage alone.^190^

Lipid accumulation in microalgae is predominantly achieved through the imposition of controlled physiological stress, most commonly *via* nitrogen or phosphorus limitation, altered salinity, or modulation of light intensity and photoperiod. These stressors redirect cellular carbon flux away from protein and pigment synthesis toward the accumulation of triacylglycerols, enhancing biodiesel-relevant lipid content. However, stress-induced lipid accumulation is inherently accompanied by reduced photosynthetic efficiency and suppressed biomass growth, creating a well-recognised trade-off between lipid fraction and net lipid productivity. To overcome this limitation, two-stage and dynamic cultivation strategies have been developed, whereby microalgae are initially grown under nutrient-replete conditions to maximise biomass, followed by a secondary stress phase to induce lipid accumulation.^191,192^ A recent experimental study by Pandey *et al.* (2025) demonstrated this approach using *Monoraphidium* sp., reporting a substantial increase in overall lipid productivity by temporally decoupling growth and lipid induction phases while maintaining culture stability under conditions relevant to large-scale cultivation.^193^ In parallel, genetic and metabolic engineering strategies aim to constitutively enhance triacylglycerol biosynthesis pathways without severe growth penalties; nevertheless, long-term genetic stability, regulatory acceptance, and performance under outdoor, non-sterile environments remain critical barriers to industrial implementation.^194^

### 7.5. Reactor design, resource integration, and scale-up constraints

Microalgal cultivation systems must reconcile three competing objectives—high biomass and lipid productivity, low capital and operating costs, and scalability under real environmental conditions. Open raceway ponds remain the most economically accessible option due to their simple design, low construction cost, and suitability for large-area deployment. They are particularly attractive for integration with wastewater treatment, where nutrient availability can partially compensate for inherently low volumetric productivity. However, open systems are fundamentally limited by poor light utilisation efficiency, evaporative water losses, susceptibility to biological contamination, and low achievable biomass concentrations, which collectively constrain areal lipid yields. Recent large-scale assessments confirm that while open ponds can support bulk biomass generation, their standalone use is insufficient for fuel-scale biodiesel production unless combined with upstream strain optimisation or downstream process intensification.^188^

Closed photobioreactors provide substantially greater environmental control, enabling precise regulation of light exposure, CO_2_ delivery, and nutrient supply, which translates into higher volumetric productivity and more consistent biomass composition. These systems are particularly effective for high-value inoculum production and for strains sensitive to contamination or fluctuating outdoor conditions. Nevertheless, photobioreactors incur significantly higher capital costs and parasitic energy demands associated with mixing, cooling, and artificial illumination, and their scale-up is constrained by hydrodynamic and thermal management challenges. Consequently, recent techno-economic and experimental studies increasingly position photobioreactors not as standalone fuel production units, but as intensification modules within hybrid cultivation schemes.^195^ For example, Tripathi *et al.* (2025) demonstrated that a two-stage strategy combining photobioreactor-based biomass amplification with downstream open-pond lipid induction substantially improved net lipid productivity while moderating system-level costs.^196^

The most promising route toward long-term microalgal biodiesel deployment lies in the integration of cultivation with wastewater treatment, saline water utilisation, or industrial effluent management. Wastewater-based cultivation supplies nitrogen and phosphorus at negligible cost while enabling simultaneous nutrient removal, thereby offsetting cultivation expenses and delivering regulatory and environmental co-benefits. Similarly, saline and industrial effluent systems reduce freshwater dependency and expand geographic deployment potential, particularly in arid and coastal regions. Life-cycle and pilot-scale studies increasingly demonstrate that such integrated systems achieve superior energy and carbon balances compared with freshwater-dependent cultivation.^197^ Liu *et al.* (2025), for instance, showed that coupling microalgal growth with municipal wastewater treatment enabled net greenhouse gas reductions and improved overall process economics, reinforcing the view that resource-integrated cultivation is a prerequisite, rather than an optional enhancement, for fuel-scale microalgal biodiesel deployment.^162^

### 7.6. Downstream processing and energy constraints in lipid recovery

Downstream processing remains the most significant energy and economic barrier in microalgal biodiesel systems, often dominating the total production cost and severely constraining commercial viability. The inherently low biomass concentration of microalgal cultures necessitates intensive harvesting and dewatering operations to concentrate cells prior to lipid extraction, with traditional centrifugation achieving high recovery efficiencies but at prohibitive energy input and operational cost. Alternative approaches such as flocculation, flotation, and membrane filtration offer reduced energy demand but involve trade-offs in recovery efficiency, chemical consumption, and scalability. Beyond concentration, cell disruption techniques—including mechanical milling, high-pressure homogenisation, ultrasonics, and pulsed electric field pretreatment—are required to breach rigid algal cell walls to access intracellular lipid pools. Each of these methods contributes additional energy penalties, and their integration with subsequent extraction steps often results in cumulative downstream energy consumption that can exceed the energy content of the recovered lipid. A recent systematic review highlights the critical role of harvesting and extraction technologies as bottlenecks in microalgal biodiesel production, citing the need for integrated, low-energy process chains to meaningfully improve overall sustainability metrics.^61,198^

Lipid extraction strategies themselves exhibit complex trade-offs between efficiency, scalability, solvent use, and environmental impact. Conventional solvent-based methods (*e.g.*, Bligh & Dyer or Soxhlet) achieve high lipid recovery but entail hazardous solvents, high solvent volumes, and energy-intensive solvent recovery/distillation steps. Emerging techniques such as biphasic lipid extraction following pulsed electric field (PEF) pretreatment have demonstrated substantial reductions in energy demand by enhancing cell permeability and reducing solvent use, with Papachristou *et al.* (2025) reporting that PEF combined with biphasic ethanolic extraction can reduce downstream extraction energy by nearly an order of magnitude compared to monophasic routes.^199^ At the same time, supercritical fluid extraction (*e.g.*, scCO_2_) and ionic liquid-assisted extraction are gaining traction for their potential to combine high selectivity with reduced environmental impact, though they currently face scale-up and equipment cost challenges. Integrative approaches that combine targeted cell disruption with low-energy extraction agents or hybrid solvent systems are increasingly recognised as essential for overcoming downstream processing barriers and realising net-positive energy balances in industrial microalgal biodiesel production.^200^

### 7.7. Critical assessment of commercialization challenges and industrial feasibility

Despite the considerable advantages of microalgae as a third-generation biodiesel feedstock, their large-scale commercial deployment remains constrained by substantial techno-economic and engineering challenges. While microalgal systems offer exceptional areal lipid productivity, carbon capture capability, and independence from arable land, these benefits do not automatically translate into economic competitiveness. Cultivation infrastructure, particularly closed photobioreactors, requires significant capital investment, while harvesting, dewatering, cell disruption, and lipid extraction collectively account for a major proportion of total production costs and energy consumption. Furthermore, maintaining stable biomass productivity under outdoor conditions remains challenging due to contamination risks, fluctuating environmental conditions, and variable carbon and nutrient availability. Recent techno-economic assessments consistently indicate that biodiesel production from microalgae remains substantially more expensive than conventional biodiesel derived from vegetable oils or waste lipids when fuel is considered the sole product. Consequently, the commercial viability of microalgal biodiesel is increasingly linked to integrated biorefinery concepts that combine fuel production with wastewater treatment, carbon capture, nutrient recovery, and the valorisation of high-value co-products. Therefore, future success will depend not only on improvements in biological productivity but also on reducing downstream processing costs, enhancing process integration, and achieving favourable life-cycle and economic performance at industrial scale.^61,201,202^

## 8. Techno-economic analysis and life-cycle sustainability of advanced biodiesel systems

Techno-economic analysis (TEA) is indispensable for assessing the industrial feasibility of advanced biodiesel systems, as it explicitly links laboratory-scale performance to capital and operating cost structures. Across both terrestrial and microalgae-based biodiesel platforms, production costs are governed not only by conversion efficiency but also by capital expenditure (CAPEX) associated with cultivation infrastructure and operating expenditure (OPEX) arising from harvesting, dewatering, lipid extraction, and downstream processing. Recent TEA studies indicate that cultivation, harvesting, and biomass concentration can account for approximately 60–80% of total production costs in microalgal biodiesel systems, with cultivation and dewatering alone frequently contributing more than 50% of the minimum fuel selling price (MFSP). In contrast, catalyst-related expenditures generally represent a comparatively smaller fraction of OPEX. Sensitivity analyses consistently identify biomass productivity, downstream energy consumption, and catalyst lifetime as the dominant determinants of economic performance. Although microalgae offer exceptional areal productivities, non-arable land utilization, and carbon-capture potential, reported MFSP values for stand-alone microalgal biodiesel systems commonly remain within the range of approximately 3–8 US$ per L, substantially exceeding those reported for waste-derived biodiesel pathways (typically 0.8–1.5 US$ per L). Consequently, current TEA studies emphasize that large-scale commercialization of microalgal biodiesel will depend on process intensification, energy-efficient harvesting technologies, catalyst durability, and integrated system-level optimization rather than improvements in conversion chemistry alone.^214–217^

Integrated biorefinery concepts have therefore emerged as a central strategy for improving economic robustness and mitigating market risk through the distribution of costs across multiple value streams. Coupling biodiesel production with the valorisation of high-value co-products—including proteins, pigments, antioxidants, fertilizers, nutraceuticals, and specialty bio-based chemicals—can substantially reduce effective fuel costs while enhancing investment resilience. This approach is particularly important for microalgal systems, where fuel-only production pathways rarely achieve economic viability under current market conditions. Recent TEA studies indicate that co-product revenues can offset a significant proportion of cultivation and processing expenses, in some cases reducing the effective production cost by 20–50%, thereby transforming biodiesel from a stand-alone fuel product into one component of a broader circular bioeconomy framework. Furthermore, integrated biorefineries reduce vulnerability to feedstock price fluctuations and policy uncertainty while improving resource utilization through nutrient recycling, wastewater treatment, and carbon dioxide valorization. These advantages collectively enhance investment resilience and strengthen the long-term attractiveness of advanced biodiesel technologies for industrial deployment and commercial investment.^21,218–220^

Life-cycle assessment (LCA) provides the complementary sustainability perspective required to inform technology selection and policy decisions, with outcomes strongly dependent on system boundaries, functional units, and integration assumptions. Comparative LCAs reveal that advanced biodiesel systems can achieve substantial greenhouse-gas (GHG) mitigation only when low-carbon electricity, waste-derived feedstocks, nutrient recycling, and CO_2_ utilization are incorporated into process design. Recent cradle-to-grave assessments report life-cycle GHG emissions of approximately 15–45 g CO_2_ per eq MJ for waste-derived biodiesel pathways and 10–40 g CO_2_ per eq MJ for integrated microalgal biorefineries, compared with approximately 85–95 g CO_2_ per eq MJ for conventional petroleum diesel. These values correspond to potential GHG reductions of approximately 50–90%, depending on feedstock selection, energy source, and co-product allocation methodology. In contrast, systems reliant on freshwater cultivation, fossil-derived utilities, or energy-intensive downstream processing often exhibit substantially reduced environmental benefits and, in some cases, only marginal life-cycle advantages. Recent assessments further confirm that microalgae-based biodiesel integrated with wastewater treatment and industrial CO_2_ capture can outperform both conventional biodiesel and fossil diesel in terms of carbon footprint reduction, resource recovery, and circularity metrics, whereas non-integrated configurations remain environmentally and economically less favorable.^162,221,222^

Collectively, TEA and LCA studies demonstrate that the long-term viability of advanced biodiesel systems is governed by a complex interplay between economic performance, resource efficiency, and environmental sustainability. The most favorable outcomes are consistently associated with integrated biorefinery configurations that combine biodiesel production with wastewater treatment, industrial CO_2_ utilization, nutrient recovery, and co-product valorisation. Under these conditions, both economic competitiveness and environmental performance can be substantially improved, facilitating the transition from laboratory-scale demonstrations toward practical biodiesel production systems with long-term deployment potential. These findings reinforce the importance of aligning techno-economic optimization with life-cycle performance through integrated design, transparent system boundaries, and evidence-based decision-making when guiding future industrial deployment and policy development.

## 9. Challenges and future perspectives

Despite significant advances in microalgal biodiesel research, the transition from laboratory-scale demonstrations to commercially viable production remains constrained by fundamental scale-up challenges. Although photobioreactors (PBRs) can achieve high biomass productivity under controlled conditions, industrial deployment introduces complex engineering limitations, including light attenuation, non-uniform photon distribution, oxygen accumulation, thermal gradients, biofouling, and elevated energy requirements for mixing and temperature control. These factors frequently reduce volumetric productivity while increasing both capital expenditure (CAPEX) and operating expenditure (OPEX), thereby limiting commercial competitiveness. Furthermore, many advanced cultivation technologies remain within intermediate Technology Readiness Levels (TRLs), highlighting the persistent gap between proof-of-concept demonstrations and fully integrated industrial implementation. Consequently, future progress will require integrated reactor-process co-design, advanced light-management systems, modular cultivation architectures, digital process control, and predictive scale-up frameworks capable of maintaining productivity under industrial operating conditions.^223–226^

Beyond cultivation, downstream processing remains one of the most significant barriers to large-scale commercialization. Harvesting, dewatering, drying, and lipid extraction collectively constitute the most energy-intensive and costly stages of the microalgal biodiesel value chain. Conventional technologies, including centrifugation and pressure-driven membrane filtration, are often economically prohibitive when applied to large volumes of dilute algal suspensions. Emerging approaches based on bioflocculation, electrocoagulation, gravity-assisted settling, magnetic separation, and hybrid membrane systems show considerable promise for reducing energy demand; however, their long-term operational stability and industrial scalability remain insufficiently validated. Equally important is the development of integrated downstream platforms capable of simultaneously recovering biodiesel precursors and high-value coproducts, thereby transforming biomass processing from a cost-intensive operation into a value-generating component of a circular biorefinery framework.^227–230^

From a systems perspective, these technical limitations directly influence techno-economic performance, environmental sustainability, and market competitiveness. Recent assessments consistently identify downstream energy demand, lipid extraction efficiency, feedstock logistics, and biomass productivity as dominant drivers of minimum fuel selling price (MFSP) and carbon intensity. In addition, large-scale implementation must contend with feedstock variability, supply-chain complexity, water and nutrient management, and infrastructure integration challenges. Although microalgal biodiesel can achieve substantial greenhouse-gas reductions when coupled with wastewater treatment, industrial CO_2_ utilization, and resource-recovery strategies, these benefits are highly dependent on process integration and operational efficiency. Therefore, future evaluations should increasingly rely on pilot- and demonstration-scale data supported by probabilistic techno-economic analysis (TEA), life-cycle assessment (LCA), and uncertainty quantification rather than idealized laboratory assumptions.^221,231,232^

Catalytic conversion technologies represent another critical determinant of industrial feasibility. While homogeneous alkaline catalysts remain commercially dominant due to their rapid reaction kinetics and low cost, their sensitivity to water and free fatty acids limits compatibility with many low-cost algal feedstocks. Alternative approaches, including heterogeneous catalysts, bifunctional catalytic systems, enzymatic processes, and supercritical alcohol transesterification, offer advantages in feedstock flexibility and downstream separation; however, challenges associated with catalyst deactivation, regeneration, leaching, operational stability, reaction rates, and process economics continue to impede widespread industrial adoption. Ultimately, overcoming these barriers will require coordinated advances in catalyst design, strain engineering, reactor technology, process integration, and policy support. Stable regulatory frameworks, carbon-pricing mechanisms, industrial symbiosis with wastewater-treatment facilities and CO_2_-emitting industries, and rigorous demonstration of long-term operational reliability will be essential for reducing investment risk and accelerating commercialization. Collectively, only a fully integrated biorefinery paradigm—combining resilient algal strains, scalable cultivation systems, low-energy downstream processing, robust catalytic technologies, and evidence-based techno-economic validation—can enable the successful transition of microalgal biodiesel from experimental promise to industrial reality.^233–236^

## 10. Conclusions

The accelerating depletion of fossil fuels, persistent carbon budget exceedance, and escalating climate-health burdens underscore the urgent need for renewable liquid-fuel alternatives compatible with existing energy infrastructures. This review has critically examined biodiesel as a strategically viable renewable fuel, establishing its environmental and performance advantages while emphasising that long-term sustainability is fundamentally governed by feedstock selection, catalytic efficiency, and systems-level integration. Through a comprehensive evaluation of feedstock evolution, the analysis demonstrates that first- and second-generation lipid sources are intrinsically constrained by land availability, resource competition, and volumetric limitations, whereas third-generation microalgal systems uniquely offer high productivity, land independence, and direct carbon capture potential. Central to advancing biodiesel beyond transitional deployment is the rational design of catalytic systems capable of operating under realistic feedstock conditions. The review highlights that catalyst–feedstock compatibility, impurity tolerance, separability, and durability are decisive determinants of industrial feasibility, particularly for waste-derived and microalgal lipids. Emerging catalytic strategies, process intensification, and advanced reactor configurations further expand the operational envelope but must be evaluated within rigorous techno-economic and life-cycle frameworks. Collectively, the findings indicate that scalable biodiesel deployment will require an integrated microalgal biorefinery paradigm that synergistically couples strain engineering, robust catalysis, low-energy downstream processing, and co-product valorisation. Such holistic integration, supported by stable policy and market frameworks, is essential to transition biodiesel from experimental promise to a resilient component of low-carbon energy systems.

## Author contributions

M. H.: conceptualization, methodology, investigation, formal analysis, visualization, writing – original draft, writing – review and editing. Y E: conceptualization, methodology, validation, supervision, writing – review and editing. P. F. F.: formal analysis, validation, writing – review and editing. W. S. E.: supervision, validation, visualization, writing – review and editing.

## Conflicts of interest

There are no conflicts to declare.

## Data Availability

Data sharing is not applicable to this article as no datasets were generated or analysed during the current study.
